# Specifically bound lambda repressor dimers promote adjacent non-specific binding

**DOI:** 10.1371/journal.pone.0194930

**Published:** 2018-04-02

**Authors:** Suparna Sarkar-Banerjee, Sachin Goyal, Ning Gao, John Mack, Benito Thompson, David Dunlap, Krishnananda Chattopadhyay, Laura Finzi

**Affiliations:** 1 Protein Folding and Dynamics Laboratory, Structural Biology and Bioinformatics Division, CSIR-Indian Institute of Chemical Biology, Kolkata, India; 2 Department of Mechanical Engineering, University of California, Merced, CA, United States of America; 3 Physics Department, Emory University, Atlanta, GA, United States of America; University of California, Davis, UNITED STATES

## Abstract

Genetic switches frequently include DNA loops secured by proteins. Recent studies of the lambda bacteriophage repressor (CI), showed that this arrangement in which the protein links two sets of three operators separated by approximately 2.3 kbp, optimizes both the stability and dynamics of DNA loops, compared to an arrangement with just two sets of two operators. Because adjacent dimers interact pairwise, we hypothesized that the odd number of operators in each set of the lambda regulatory system might have evolved to allow for semi-specific, pair-wise interactions that add stability to the loop while maintaining it dynamic. More generally, additional CI dimers may bind non-specifically to flanking DNA sequences making the genetic switch more sensitive to CI concentration. Here, we tested this hypothesis using spectroscopic and imaging approaches to study the binding of the lambda repressor (CI) dimer protein to DNA fragments. For fragments with only one operator and a short flanking sequence, fluorescence correlation spectroscopy measurements clearly indicated the presence of two distinct DNA-CI complexes; one is thought to have a non-specifically bound CI dimer on the flanking sequence. Scanning force micrographs of CI bound to DNA with all six operators revealed wild-type or mutant proteins bound at operator positions. The number of bound, wild-type proteins increased with CI concentration and was larger than expected for strictly specific binding to operators. In contrast, a mutant that fails to oligomerize beyond a dimer, D197G, only bound to operators. These data are evidence that CI cooperativity promotes oligomerization that extends from operator sites to influence the thermodynamics and kinetics of CI-mediated looping.

## Introduction

When the λ bacteriophage infects the *E*. *coli* bacterium, it propagates in either a lytic (virulent) or lysogenic (quiescent) mode. In the absence of threats to the bacterium such as poisoning, starvation or DNA damage, lysogeny is established and stably maintained by the λ repressor (CI) despite the fact that during the cell cycle, and through cell division, CI concentration varies ten-fold [1]. Nevertheless, switching to lysis is very efficient when the viability of the host is threatened. The fundamental element that allows CI to stabilize lysogeny without compromising the capability to quickly switch to lysis is a dynamic DNA loop.

**Fig 1:**
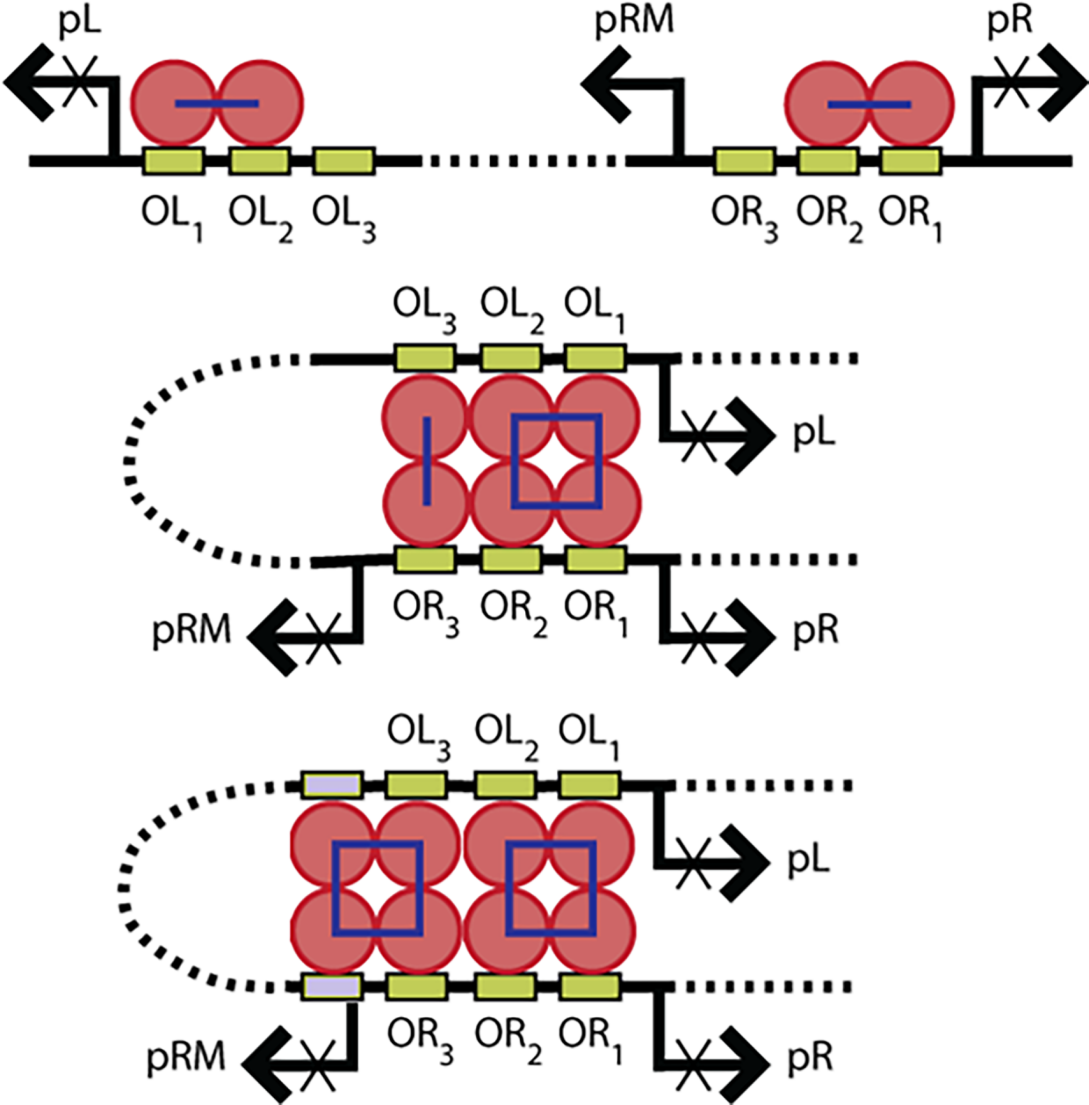
Regulation of bacteriophage gene expression by CI. (Upper) Tetramers, formed through lateral interactions of adjacent CI dimers (red circles) bound to high affinity “operator” (green) sites, inhibit transcription from the lytic promoters *pL* and *pR*. (Middle) The dimers bound at *OL*1 and *OL2* may interact head-to-head with those at *OR1* and *OR2*, to stabilize a DNA loop (dotted curve) to juxtapose and recruit dimers at the strong *OL3* and weaker OR3 sites which also interact head-to-head. The additional dimer on OR3 inhibits transcription from *pRM*. (Lower) The CI dimers at *OL3* and *OR3* are still available for lateral pairwise interaction and may cooperatively stabilize other dimers to adjacent, non-specific (gray) sites.

The CI dimer binds specifically to each of three high-affinity sites, “operators”, arranged in two sets, OL1-3 and OR1-3, in the immunity region of the lambda bacteriophage (Fig 1, green bars). Binding to the OR3 site is significantly weaker than to the other sites [1], but pairs of adjacent dimers may cooperatively interact along the DNA to form tetramers (Fig 1 top). Such cooperativity is critical for lysogeny in wild-type lambda bacteriophage [2, 3]. Tetramers or dimers at OL can also interact head-to-head with tetramers or dimers at OR to stabilize a DNA loop of approximately 2300 bp (Fig 1 center). The loop juxtaposes OL3 and OR3 to favor occupancy of this weakest operator and repress the *pRM* promoter in order to prevent an over-accumulation of CI which may interfere with switching [4, 5]. With sufficient, but not excessive, CI levels, threat-triggered degradation of the CI protein through RecA-mediated self-cleavage [1, 6] and/or a loss of supercoiling can destabilize the loop to favor the dissociation of CI and efficiently shift λ gene expression from lysogenic to lytic proteins [7].

For robust sensitivity, the CI-induced DNA loop should remain sensitive to feedback to allow switching across a range of CI concentrations. Accurate modeling of looping dynamics and DNA shortening as a function of CI concentration was found to require not only specific, but also non-specific CI-binding that facilitated loop formation and interfered with loop breakdown [8]. These concentration-dependent adjustments to the wild-type loop dynamics preserve the frequency of loop rupture, a state in which CI is vulnerable to degradation, even as the concentration of CI increases. However, a laterally unpaired dimer appears to be required, since DNA molecules with mutated OL3 and OR3 sites exhibited looping kinetics that could be modeled without considering non-specific binding.

Non-specific binding also produced end-to-end contraction of wild-type DNA as the concentration of wild-type CI increased. This is consistent with careful studies of non-specific binding in the absence of operators as a function of DNA length and other physical parameters [9]. As noted for looping kinetics, DNA molecules with mutated OL3 and OR3 sites behaved differently from wild-type and did not contract as much as CI increased. Thus, CI dimers bound to O3 sites appear to not only add a stabilizing head-to-head CI tetramer bridge to the loop closure but also seem to recruit non-specifically bound dimers through lateral interactions (Fig 1 bottom, gray bars). Although non-specific binding to DNA containing operators has previously been observed [10], it was never carefully studied, nor connected to facilitated binding via pair-wise interaction with a specifically bound CI dimer.

The assembly and oligomerization of a number of CI dimers greater than those that can be bound specifically is presently supported only by a handful of SFM images that show loop stabilizing particles of volumes consistent with two CI octamers (see Fig 6 in [11]). To conclusively test this hypothesis, fluorescence correlation spectroscopy (FCS) [12] and scanning force microscopy (SFM) were employed to assess the mass and/or shape of macromolecular complexes. FCS measurements of the diffusion of fluorescently labeled DNA oligomers containing a specific binding site for CI and enough flanking sequence for the binding of no more than one additional CI dimer, were analyzed as a function of the concentration of CI and competing, non-fluorescent oligomers. In addition, SFM studies of the volumes of CI complexes securing 400 bp loops in DNA were analyzed as a function of CI concentration. These studies show that, specifically-bound CI dimers can stabilize adjacent, non-specific binding of additional dimers, even at the weak OR3 site.

## Materials and methods

### Reagents and chemicals

All chemicals and reagents used for the expression and purification of the proteins were of molecular biology grade. Chemicals used for the fluorescence correlation spectroscopy experiments were of ultra-pure spectroscopy grade. All SFM and FCS experiments were carried out at room temperature in 10 mM Tris-HCl buffer (pH 7.4), containing 200 mM KCl, 1 mM EDTA, and 1 mM DTT.

### DNA constructs and proteins

All DNA fragments used in the FCS measurements were 60 bp-long (Integrated DNA Technologies, Coralville, USA). They included a fluorescent Alexa-488 end-label spaced five base pairs from a wild-type OL1, OL3, or OR3 operator, flanked by 38 base pairs of DNA. Depending on the experiment, the flanking DNA included 38 bp of randomly selected sequence with 68% GC content (GCRndm) or *wild-type* (wild) sequence bordering the corresponding operator externally (in the direction opposite OL2 or OR2). Each fragment was synthesized with and without the Alexa488 label, and the sequences of these DNA constructs are reported in S1 Table. In all FCS experiments, 100 nM Alexa488-labeled DNA was used.

For SFM imaging, 1555 bp-long DNA constructs were produced as described previously [11] by PCR using plasmid pDL944 [4] as a template. OR and OL were 395 bp (132 nm) and 719 bp (240 nm), respectively, from opposite ends of the construct with 401 bp (148 nm) in between.

The *wild type* (WT) and mutant (D197G) lambda repressor (CI) proteins were expressed and purified as previously reported [13], and likely have activities of at least 50% [14, 15]. Protein concentrations (both WT and D197G mutants) were varied between 0 and 500 nM for FCS or 200 and 800 nM for SFM experiments.

### Fluorescence correlation spectroscopy (FCS) experiments

FCS experiments were carried out using a Zeiss 510 META Confocor3 LSM instrument (Carl Zeiss, Evotech, Jena, Germany) with a 40X water immersion objective. A 30 mW, 488 nm argon-ion laser was set at 5% output. The number of molecules in the confocal volume was maintained between 5 and 10.

The concentrations of salt (KCl) and DNA were optimized in preliminary experiments (S1 Text and S2 Fig). Then, the diffusion of 100 nM of Alexa488-labeled DNA fragments was measured in the presence of lambda repressor protein at concentrations from 0–500 nM. For the competitive binding experiments, the diffusion of 100 nM Alexa488-labeled DNA fragments plus 500 nM lambda repressor was measured in the presence of increasing concentrations of unlabeled DNA fragments.

### FCS data analysis

For a single diffusing species (e.g. free fluorescently-labeled DNA in buffer), the autocorrelation function, *G(τ)*, is well modeled as

$$G(\tau )=1+\frac{1}{N}.\frac{1}{\left(1+\frac{\tau }{\tau_{D}}\right)}.\frac{1}{{\left(1+S^{2}\frac{\tau }{\tau_{D}}\right)}^{1/2}}$$ [1]

in which *N* is the average number of molecules in the observation volume, *S* defines the depth-to-diameter ratio of the Gaussian observation volume, *τ* is the observation time, and *τ*_*D*_ is the time for which free DNA diffuses within the observation volume. When multiple diffusive species are present (e.g. free DNA as well as DNA bound by protein), the correlation function contains additional terms:

$$G(\tau )=1+\frac{1}{N}.\sum_{i}\frac{a_{i}}{\left(1+\frac{\tau }{\tau_{Di}}\right)}.\frac{1}{{\left(1+S^{2}\frac{\tau }{\tau_{Di}}\right)}^{\frac{1}{2}}}$$ [2]

Here *τ*_*Di*_ is the diffusion time of the *i*^*th*^ diffusing species in solution and *a*_*i*_ is the relative amplitude, or fraction of the *i*^*th*^ species. The *a*_*i*_ amplitudes are normalized to satisfy:

$$\sum_{i}a_{i}=1$$ [3]

Initially, all autocorrelation functions obtained in the presence of different protein concentrations were fit using a single component diffusion model (Eq 1). However, the diffusion times, τ_D_, determined from single component fitting of autocorrelation data, plotted versus protein concentration (S3 Fig), indicated biphasic binding of CI to DNA (see detailed description of fitting strategy in S2 Text). Also, analysis of the residual fitting errors between the FCS data and autocorrelation functions indicated the need for a more complex analysis (see below). With 0, 250, or 500 nM CI protein, the FCS data for Alexa488-labeled DNA fragments could be well fit with Eq 1 including just one diffusive species (S4 Fig). However, the data in the presence of protein, at intermediate concentrations (125 and 375 nM), had to be fit using Eq 2 with terms representing two diffusive species (S5 Fig). A detailed description of the strategy for fitting the FCS data obtained for complexes between the OL1-wild DNA construct (see S1 Table) and increasing concentrations of wild-type CI protein is provided in S1 Text and S6 Fig.

**Fig 2:**
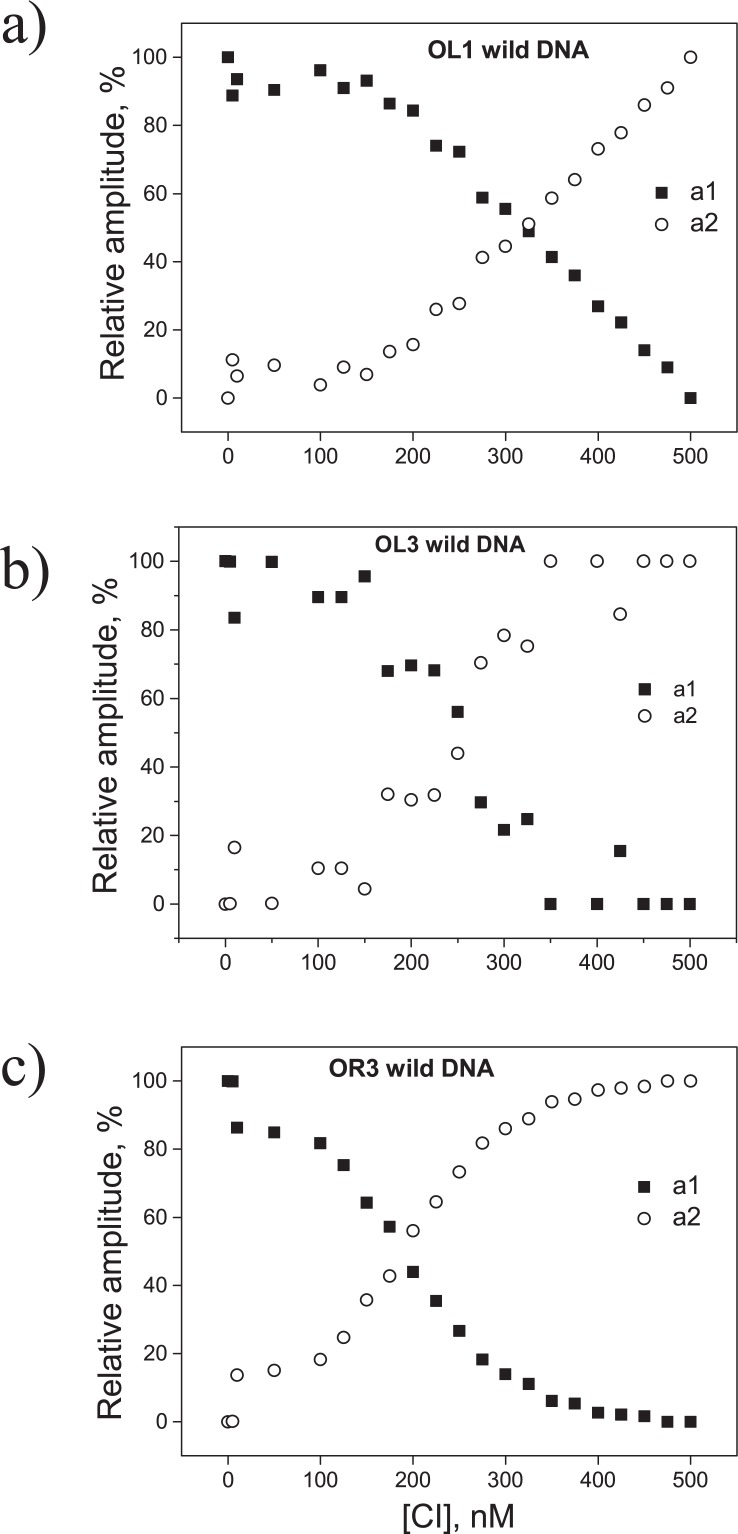
Variation in the amplitude of the mutant CI protein-DNA complex. The amplitudes of *a1* (filled squares) and *a2* (open circles) used to fit the auto-correlation data (Eq 2) are plotted as a function of mutant CI protein (D197G) concentration for (*a*) WT OL1, (*b*) WT OL3, or (*c*) WT OR3 DNA. For each DNA, *a*_*1*_ dropped from an initial maximum to a final minimum with a reciprocal rise in *a*_*2*_ as [CI] increased from 0 to 500 nM. In contrast to the wild-type protein, no third species developed at higher concentrations.

A similar fitting protocol was used for OL3-wild (S1 Table) in the presence of the D197G mutant. However, a progressive decrease in the diffusion time was observed for OL1-wild (S7 Fig) and OR3-wild (not shown) in the presence of increasing concentrations of the D197G mutant CI. We established that this decrease was not due to either free dye or triplet state photophysics. The average lifetime of the triplet state was independently determined to be 3 microseconds in these experimental and laser power conditions (data not shown), which is orders of magnitude less than the time constants for the measured auto-correlation functions (S1 Fig). In addition, the characteristic residence time of the free dye (approximately 30 μs), also independently measured in the same experimental conditions, was less than the characteristic residence time observed for labeled DNA in the presence of saturating concentrations of protein (S2 Table). We observed that an exponential component (with time constants of ~71.49 μs and ~41 μs for OL1-wild and OR3-wild, respectively) fit the data well (S1 Fig, S2 Table). As a result, in Fig 2A and 2C, the reported amplitudes, *a*_*i*_, are those corresponding to exponential components with the indicated characteristic residence times. Although, we cannot definitively describe the mechanism, these exponential forms might indicate that the mutant protein induces rapid conformational fluctuations in OL1-wild and OR3-wild.

## Results and discussion

### D197G binding characteristics

The single-point mutation of the CI repressor, D197G, fails to display cooperativity. It was characterized in [16], used in negative control measurements on the effect of CI-induced looping in transcription assays [4], and used to obtain the crystallographic structure of a lambda CI dimer bound to operator in [17]. Fig 2A–2C reveal a previously undetected feature of D197G: it binds more strongly to OR3 than to OL1 and OL3, contrary to WT CI. This is more directly seen in S8 Fig. which reports the fraction of bound protein (directly corresponding to the relative amplitude of the FCS signal as reported, for example in Fig 2) as a function of protein concentration. The dissociation constants for the two proteins from each operator type are provided in S3 Table. Note that the inverse affinity pattern of D197G for the lambda operators does not affect the conclusions of this work.

### FCS data reveal two distinct DNA-CI complexes

**Fig 3:**
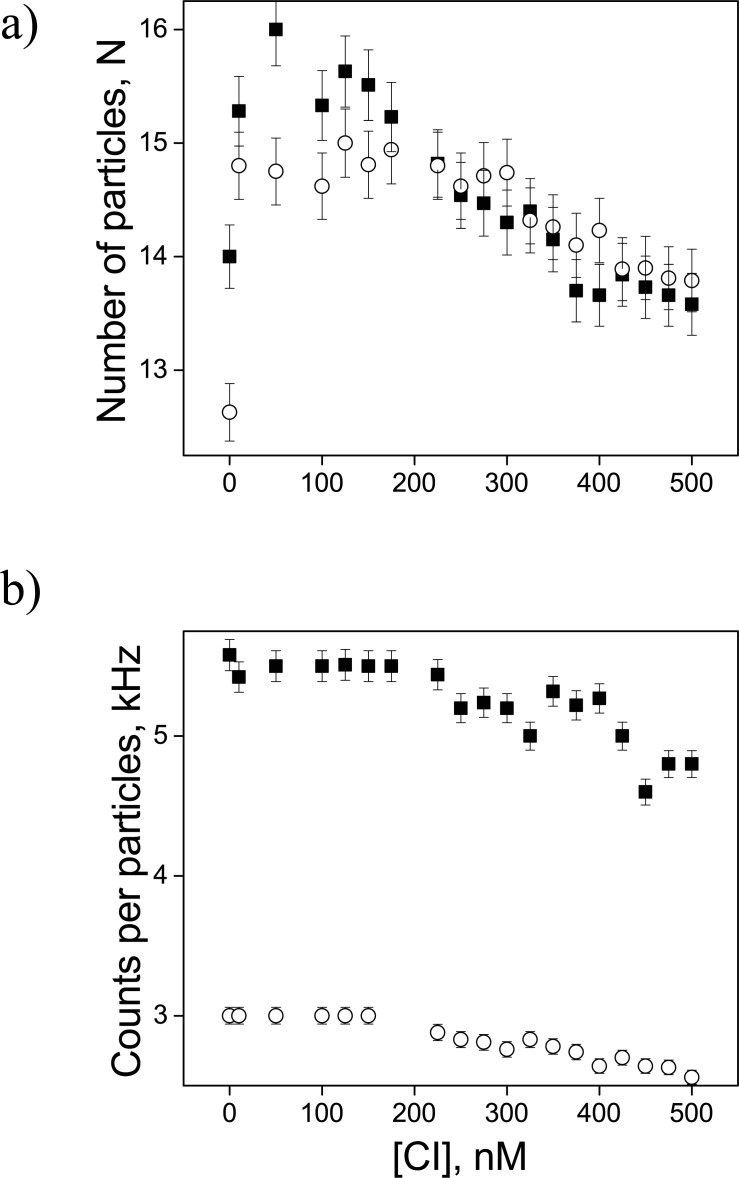
**Variations in the numbers of particles (*a*) and counts per particle (*b*) as a function of CI protein concentration.** Squares (filled) and circles (open) correspond to WT DNA constructs containing the OL3 or OR3 sites, respectively.

After optimizing the experimental conditions (S1 Text), we studied the binding between the WT protein and the fluorescently labeled DNA as a function of CI concentrations (Fig 3A and 3B). The autocorrelation functions revealed negligible variation in the number of particles, and especially counts per particle, and no indication of the formation of “sandwich” complexes, in which two CI dimers, interacting pairwise through C-terminal domains, connect two Alexa-488-labeled DNA molecules.

**Fig 4:**
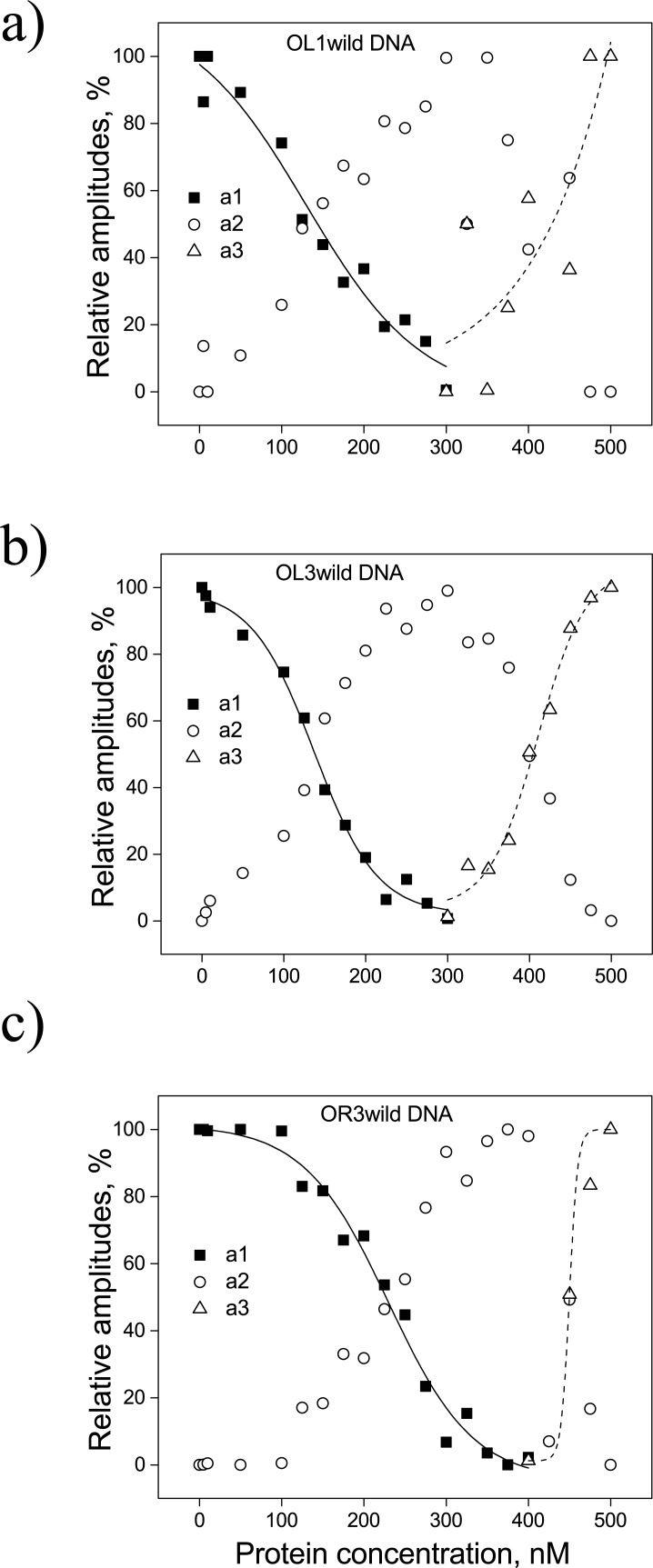
Variation in the amplitude of the WT CI protein-DNA complex. The three amplitudes *a*_*1*_ (filled square), *a*_*2*_ (open circle), and *a*_*3*_ (open triangle) used to fit the auto-correlation data (Eq 2) are plotted as a function of CI protein concentration for (*a*) WT OL1, (*b*) WT OL3, or (*c*) WT OR3 DNA. Solid and dashed curves serve as guides for the transitions of *a*_*1*_ and *a*_*3*_ as the concentration of CI changes. For each DNA, *a*_*1*_ dropped from an initial maximum to a final minimum with a reciprocal rise in *a*_*2*_ as [CI] increased from 0 to 300 nM (*a*, *b*) or to 400 nM (*c*). As [CI] was increased further, *a*_*2*_ decreased to a minimum and *a*_*3*_ rose to maximum values at 500 nM CI.

These autocorrelation functions were fit using either a one-, or a two-component model (Eqs 1 or 2) since, of the three possible species, only one or two were present at any given CI concentration (strategy described in S2 Text). The variation of the different species amplitudes (*a*_*1*_, *a*_*2*_, and *a*_*3*_) obtained with DNA constructs containing OL1, OL3, or OR3 in the presence of different concentration of WT CI protein are shown in Fig 4A–4C.

Fig 4A shows that the correlation function data of the free OL1-wild DNA (CI concentration = 0 nM) was well fit by a single diffusive component (*a*_*1*_ = 1, *a*_*2*_ = 0) corresponding to free DNA. With the addition of protein, the amplitude of *a*_*1*_ decreased as *a*_*2*_ simultaneously increased. At a CI concentration of approximately 300 nM, the value of *a*_*1*_ became zero and *a*_*2*_ reached a maximum. As more protein was added, the value of *a*_*2*_ also started to decrease, and *a*_*3*_ increased. Similar trends were observed when WT CI was added to the other DNA constructs (Fig 4B and 4C). This behavior is consistent with the existence of free DNA in the absence of protein and with the progressive formation of a primary DNA-CI species, as the CI concentration initially increases. When the concentration raises enough to eliminate free DNA, a secondary, higher order CI complex begins to form. Therefore, the interaction between WT CI protein and any of the DNA constructs could be represented by a series of reactions:

$$Free\;DNA+CI↔{\left(DNA-CI\right)}_{c1}{\cdots (DNA-CI)}_{c1}+CI↔{\left(DNA-CI\right)}_{c2}$$

in which the subscripts *c1* and *c2* indicate type 1 or type 2 DNA-CI complexes, respectively. To analyze the oligomerization state of CI in these complexes, we assumed that the two complexes had *n*_*1*_ and *n*_*2*_ numbers of CI dimer proteins, respectively.

### Determination of n_1_ and n_2_ indicates specific, followed by non-specific, binding

The numbers of CI dimers bound to DNA in the *c1* and *c2* complexes were derived by calculating the hydrodynamic radii, *r*_*H*_, of the two types of complexes, estimating their molecular masses, and recognizing the fact that molecular weights of a CI dimer and the 60 bp DNA fragment are nearly the same, (approximately 40 kDa).

Using Hydropro modelling [18] and the crystal structure of the protein bound to a DNA operator site [17], the average radius of the lambda dimer (*r*_*c*_) was calculated to be 28.7 Å. This value is in excellent agreement with previously reported radii, 25.2 Å [19].

For either of the two possible DNA-protein complexes, the length of the 60 bp DNA, ≃0.34 nm×59 ≃ 20.0 nm (200 Å), is more than twice the thickness of DNA (20 Å) and a CI-dimer combined. Therefore, the hydrodynamic radii of the DNA-CI complexes were calculated as follows by approximating them as cylinders.

First, the diffusion times, *τ*_*D*_, obtained from the FCS data were used to calculate the diffusion coefficients (*D*) of a labeled DNA-protein complexes using the equation,

$$\tau_{D}=\frac{\omega^{2}}{4D}$$ [4]

in which *ω* is the axial radius of the light beam used in the FCS apparatus to illuminate the sample. Next, the effective hydrodynamic drag (*γ*) was calculated from the diffusion coefficient, *D*, using the Stokes’ Einstein relation,

$$\gamma =\frac{kT}{D}$$ [5]

in which *k* is the Boltzman’s constant and *T* is the absolute temperature. The effective drag, of cylindrical particles was approximated by their axial drag coefficients only (ignoring lateral drag), which for finite-length cylindrical objects can be derived from Navier-Stokes equations [20] as

$$\gamma =\frac{2\pi \eta L}{ln\frac{L}{2r_{H}}-0.20},$$ [6]

in which η is the viscosity of water (0.89 cP), *L* is the length of the cylinder (DNA), and *r*_*H*_ is the hydrodynamic radius. The values of *r*_*H*_ obtained using Eq (6) for CI-DNA complexes containing OL1, OL3 or OR3 were 16.6, 18.8 and 19.5 Å, or 20.9, 20.3, and 21.5 Å for types *c1* and *c2* respectively (Table 1).

**Table 1:** Number of CI dimer that bind during the first (*n*_*1*_) and second (*n*_*2*_) transitions.

Protein	DNA constructs	*r*_*H*_ for (DNA-CI)_c1_ (Å)	*r*_*H*_ for (DNA-CI)_c2_ (Å)	*n*_*1*_	*n*_*2*_
Wild-type	OL1-wild	16.6	20.9	0.75	1.78
Wild-type	OL3-wild	18.8	20.3	1.24	1.61
Wild-type	OR3-wild	19.5	21.5	1.42	1.94
Wild-type	OL1-GCRndm	16.0	21.4	0.62	1.89
Wild-type	OL3-GCRndm	15.4	18.6	0.50	1.20
Wild-type	OR3-GCRndm	14.0	nd.	0.25	—
Wild-type	OL3-wild	18.8	20.3	1.24	1.61
D197G	OL1-wild	anomalous*	nd.	—	—
D197G	OL3-wild	14.8	nd.	0.39	—
D197G	OR3-wild	anomalous*	nd.	—	—

*Reasonable estimates of *r*_*H*_ values could not be established for OL1 and OR3 in the presence of D197G, due to anomalously fast diffusion of these complexes. “nd” indicates “not detected”.

Since the molecular masses of these DNA-CI complexes are proportional to their volumes, and using the radius of the lambda dimer (*r*_*c*_) of 28.7 Å (see above) to estimate the volume of a CI dimer, we calculated the ratio between the molecular mass of the complex, (DNA-CI)_*c1*_ or (DNA-CI)_*c2*_, *M*_*(c1 or c2)*_, and that of the CI dimer, *M*_*c*_:

$$\frac{M_{\left(c1\;or\;c2\right)}}{M_{c}}=\frac{\pi {{r_{H}}_{\left(c1\;or\;c2\right)}}^{2}L}{\frac{4}{3}\pi r_{c}^{3}}$$ [7]

*M*_*(c1 or c2)*_ in this equation includes the mass of the 60 bp-long DNA fragment which has a molecular weight equivalent to that of the CI dimer. Therefore, by subtracting one from these ratios, we estimated the number of dimers, *n*_*1*_ or *n*_*2*_, present in (DNA-CI)_*c1*_ or (DNA-CI)_*c2*_ complexes, respectively,

$$n_{1}=\frac{M_{1}}{M_{c}}‑1\;\mathrm{and}\;n_{2}=\frac{M_{2}}{M_{c}}‑1$$ [8]

For operators with adjacent wild-type sequence, the first complex to form, as the CI concentration was raised, appears to include approximately one dimer, probably specifically bound (Table 1). A further increase in the concentration produced a more slowly diffusing complex containing approximately two dimers. Given the purposefully limited, specific binding platform of the DNA fragments, it is reasonable to conclude that this second dimer was non-specifically bound. For operators with an adjacent random sequence of a high GC content, the hydrodynamic radii were smaller and led to lower estimates of the number of dimers in the first species to develop, *n*_*1*_. Nonetheless, the hydrodynamic radii measured for the second species were larger, consistent with the presence of an additional dimer on the DNA constructs OL1-GCRndm and OL3-GCRndm. For the lower affinity sequence OR3-GCRndm DNA, *n*_*1*_ was very low and a second, more slowly diffusing, species could not be detected. The same was true for experiments performed with the mutant CI dimer, D197G, which cannot interact pairwise with an adjacent dimer [16]. In this case the (DNA-CI)_*c2*_ species was not observed (Fig 2), and only *r*_*H*_ values for (DNA-CI)_*c1*_ complexes could be calculated.

Fitting the auto-correlation functions for the diffusing particles as mixtures of species produced estimates of the component fractions of each species as a function of CI concentration. CI (wild-type) concentrations at which amplitudes *a*_*1*_ and *a*_*2*_, or *a*_*2*_ and *a*_*3*_ are equal (only *a*_*1*_ and *a*_*2*_ with mutant D197G, Fig 2) are listed as mid-points in Table 2. They indicate a transition between prevalent types of DNA-CI complexes during CI titrations. As evident from Table 2 and Fig 4, wild-type protein binds to the OL3 and OL1 DNA constructs with similar affinities and generates similar transitions points. This is in contrast to OR3, which binds more weakly and exhibits a shifted transition point. The mid-points of the second transitions were approximately similar for all the operator fragments (Table 2). The transition points for CI protein binding to GC-rich, random sequences (OL3-GCRndm DNA and OL1-GCRndm DNA) were very similar to those measured for OL3-wild DNA and OL1-wild DNA (Table 2). For the OR3-GCRndm DNA, the first transition was similar to that for OR3-wild DNA, suggesting weak binding, while the second transition was difficult to resolve. In summary, the changes in *r*_*H*_ for the GC-rich, random sequences were calculated and found to be somewhat lower, but substantially similar to those for the wild-type DNA sequences.

**Table 2:** The CI concentrations at mid-points of the first and second transitions for titrations of CI with the indicated DNA constructs as shown in Figs 3 and 4. These are indicative measures of relative binding affinities for the different CI operators.

Protein	DNA construct	Mid-point of the first transition (nM)	Mid-point of the second transition (nM)
Wild-type	OL1-wild	129	430
Wild-type	OL3-wild	137	410
Wild-type	OR3-wild	230	460
Wild-type	OL1-GCRndm	151	378
Wild-type	OL3-GCRndm	131	377
Wild-type	OR3-GCRndm	217	—
D197G	OL1-wild	325	—
D197G	OL3-wild	255	—
D197G	OR3-wild	191	—

**Fig 5:**
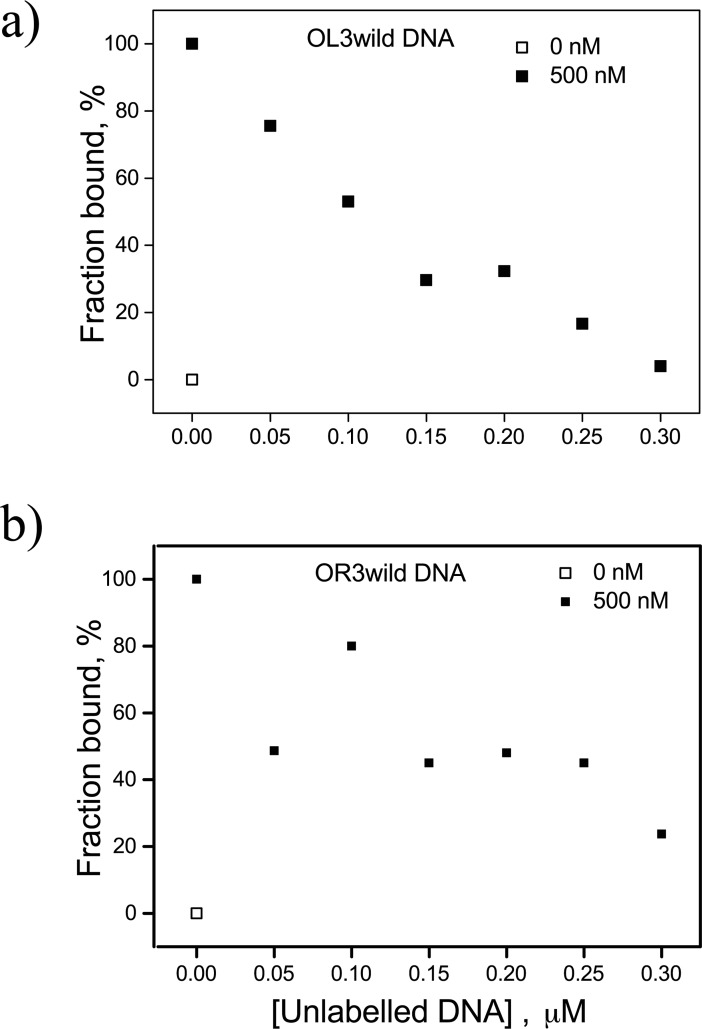
**Titrations of unlabeled DNA to assay competitive CI-binding to (*a*) wild-type OL3 or (*b*) wild-type OR3 DNA.** Increasing the concentration of unlabeled DNA (filled squares) from 0 to 0.3 μM in the solution used for FCS measurements reduced the fraction of CI bound. This decrease was characterized by a plateau for both OL3 and OR3. Clearly, competition by the unlabeled DNA progressively reduced the CI available for binding to the fluorescently labeled DNA. This is evidence against the presence of higher molecular weight complexes in which a head-to-head interaction of CI dimers (Fig 1) might stabilize FCS particles containing a labeled and an unlabeled DNA segment.

To further support these FCS findings, we performed competitive binding experiments between labeled and unlabeled operator DNA constructs and the CI protein. In these experiments, increasing amounts of unlabeled DNA were added to a solution of fluorescently labeled DNA which was saturated with wild-type CI protein. Fig 5 clearly shows that unlabeled DNA competes for the protein which dissociates from labeled DNA. Fig 5A and 5B show the effect of unlabeled competitor DNA on OL3 and OR3, respectively. These data are consistent with the fact that OL3 has stronger affinity for the CI protein than OR3. Most interestingly, the data in both panels display what might be a plateau indicating competition with DNA fragments involved in the two species, (DNA-CI)_c1_ and (DNA-CI)_c2_, detected in the FCS measurements described above (Fig 4).

Thus, as the CI concentration increased, first, one more slowly diffusing DNA species and then another even slower species appeared as the fraction of free DNA diminished. Considering that these species are likely to be ellipsoidal, not spherical, we approximated them as cylinders. Then, using the measured diffusion coefficient and Navier-Stokes equations to calculate the hydrodynamic radii of the particles, we estimated the number of CI dimers in the two DNA-CI species (Table 1: *n*_*1*_ and *n*_*2*_). Because of the approximations used, these values are coarse estimates of *n*_*1*_ and *n*_*2*_. However, *n*_*2*_ was approximately one unit greater than *n*_*1*_. This is consistent with the idea that the DNA fragments are first bound by one CI dimer, likely specifically bound at the operator site, and later by a second dimer at the flanking, non-specific sequence. Approximating the diffusing particles as spheres yielded large hydrodynamic radii, and molecular masses, and unreasonably high values of *n*_*1*_ and *n*_*2*_ (data not shown). This supports our choice to model the DNA-CI complexes as cylinders. The counts per particle excluded the possibility of “sandwich” complexes in which two fluorescently labelled DNA molecules would have been held together by bridging CI tetramers. Also, adding unlabeled, DNA with identical sequences to solutions containing saturating amounts of wild-type CI did not produce even more slowly diffusing particles as would be expected for such complexes. Furthermore, the CI concentration used in these studies is within the physiological range and well below the concentration at which CI in solution spontaneously assembles into tetramers (1x10^-5^ M) [1, 21]; thus, we exclude that a specifically bound CI may interact with another dimer head-to-head. Therefore, the data support the idea that wild-type CI loads onto the DNA first specifically and, later, non-specifically as the protein concentration is increased. The fact that the D197G mutant, does not exhibit a secondary FCS complex, nor loop-securing aggregates that increase with concentration in nano-topographs (S9 Fig), suggests that the nonspecific binding of the second, wild-type CI dimer is likely facilitated by pair-wise interaction with the adjacent, specifically bound dimer.

Approximating the complexes as cylindrical particles in order to estimate their hydrodynamic drag may underestimate the values of *n*_*1*_ or *n*_*2*_. The uncertainties inherent to hydrodynamic modeling, due to sensitivity to the CI dimer radius and the disregard of lateral drag, can account for the large variation in the computed values of either *n*_*1*_ or *n*_*2*_ in Table 1. However, it is also plausible that the variations indicate the co-existence of several diffusive species in the FCS data. By reducing/eliminating the hydrodynamic uncertainties with more accurate computation fluid dynamics (CFD) models, variations in *n*_*1*_ or *n*_*2*_ that remain might then be used to resolve the binding affinities of the operators. In principle, CFD simulations based on the Navier-Stokes equations can take into account any shape as well as deformability of particles and yield a more accurate effective drag. However, this level of accuracy is unnecessary given the experimental noise and errors, and the ultimate goal of obtaining estimates of the integers *n*_*1*_ and *n*_*2*_ that are both distinct and compatible with a realistic interpretation. In this respect, it is straightforward to show that approximating the complexes to equivalent spherical particles having the same hydrodynamic drag tends to substantially and spuriously over-estimate values of *n*_*1*_ or *n*_*2*_.

The CI concentration at the midpoint of the transition from free DNA to the more rapidly diffusing DNA-CI species, which we interpreted as a complex containing one specifically bound CI, was similar for the two high affinity operators OL1 and OL3. However, the midpoint for OR3 occurred at a higher CI concentration, consistent with the lower affinity of CI for that operator (Table 2). In contrast, the CI concentration for the subsequent transition to a second DNA-CI species, which we interpreted as a complex containing one specifically bound CI dimer and additional one bound non-specifically to the flanking DNA sequence, was similar for all three operators. This implies that regardless of the affinity of their specific interaction, bound dimers similarly promote the non-specific binding of an adjacent dimer. Notably, the transition to the second DNA-CI complex occurred at slightly lower CI concentrations in GCRandom templates, indicating that the random, GC-enriched flanking sequence may be a better substrate for non-specific binding (Table 2).

### Loop-securing, CI complexes in scanning force nanographs grow with [CI]

**Fig 6:**
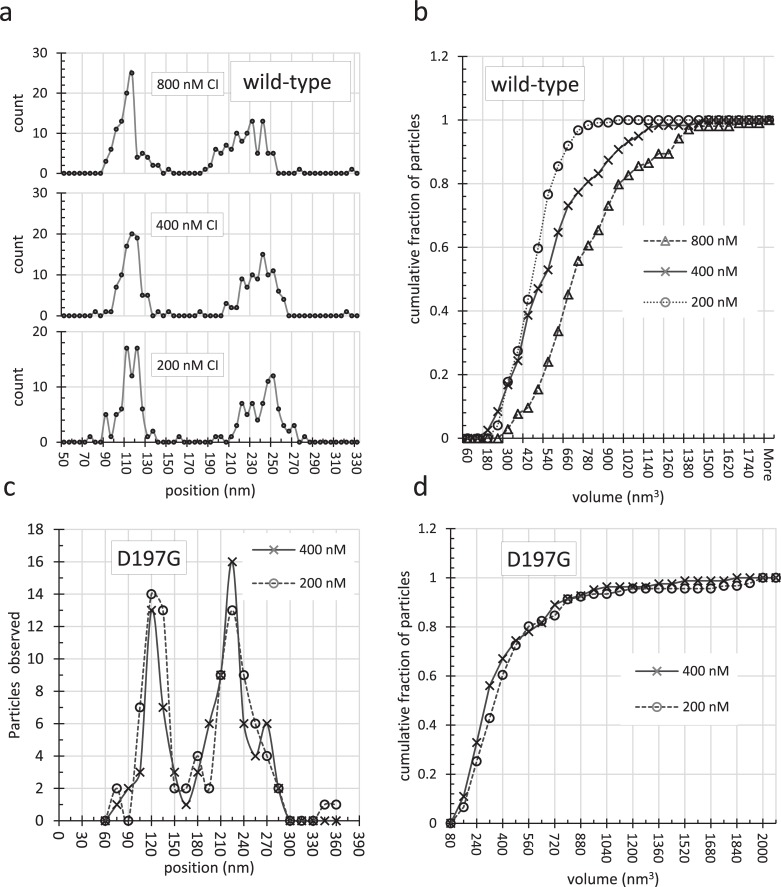
Measurement of positions and volumes for CI multimers which appeared as amorphous particles along DNA fragments containing OL and OR binding sites. (*a*) The positions of these protein particles were measured with respect to the nearest end at different concentrations. Even at the highest protein concentration almost all particles were found near 120 and 240 nanometers into the contour, where high affinity binding sites are located. (*b*) The sizes of CI particles increased with concentration as can be seen in a cumulative histogram of the volumes measured in scanning force nanographs recorded for DNA exposed to 200 (circles), 400 (crosses), or 800 (triangles) nM CI. (*c*) The D197G mutant CI protein also formed specifically positioned particles on the OL and OR bindings sites with little binding elsewhere. (*d*) However the particles of mutant protein, that does not form multimers larger than dimers, did not increase in size as a function of CI concentration.

Using SFM to directly visualize non-specific binding of a CI dimer adjacent to a dimer specifically bound at a single operator (semi-specific binder of a CI tetramer) is challenging. Due to the small size of the dimer, approximately 42 kDa, it is difficult to unequivocally distinguish a dimer from a tetramer. However, it is possible to monitor the increase in the aggregate volume of the CI complexes securing loops at different CI concentrations. DNA complexes with wild-type, or the D197G mutant CI, were imaged using scanning force microscopy. These experiments involved a 1555 bp-long DNA fragment containing the OL and OR regions separated by 401 bp. S9 Fig shows representative nanographs which were analyzed to determine the position and oligomerization state of CI binding. Both WT (Fig 6A) and D197G CI (Fig 6C) bound specifically to operators but the volume of the WT-DNA complex increased with CI concentration (Fig 6B), while the D197G-DNA complex did not (Fig 6D).

The increase in the volume of the wild-type CI complex securing the loop appears to indicate the binding of more dimers than expected based on the number of specific sites. In previously published calibration curves, particle volumes of 400, 600, or 800 nm^3^ were correlated with complexes of 6–8, 10–12, or 14–16 monomers [11]. In the present work, at 200 nM CI only 15% of loop-securing complexes were larger than 600 nm^3^, the maximum expected for strictly specific binding (Fig 6B). Further increasing the CI concentration to 400 and 800 nM increased this fraction to 36 and 67% respectively. In addition, the maximum volume observed increased from 780 nm^3^ at 200 nM CI to 1080 and 1320 nm^3^ at 400 and 800 nM CI respectively. These larger values correspond to complexes 125–200% of the estimated volume of a specific CI complex composed of 10–12 monomers. This is in agreement with a previous theoretical prediction that the number of proteins securing a DNA loop increases with the number, N, of protein binding sites and with protein concentration, although that work did not consider pairwise cooperativity explicitly as a means for increasing the effective number of binding sites [22].

In contrast, dimers of D197G, which cannot interact pairwise, only bind to specific sites and the volume of the mutant protein-DNA complex did not increase with protein concentration (Fig 6D). Approximately 18% of CI complexes were larger than 600 nm^3^ and increasing the protein concentration to 400 nM had no effect. Thus the mutant protein did not appear to stabilize adjacent dimers on flanking DNA. This result is consistent with our hypothesis that a specifically bound dimer stabilizes another dimer at an adjacent non-specific sequence via lateral, pair-wise interaction.

It is important to note that several previously reported footprinting assays of the binding of the lambda CI protein to short fragments of DNA containing single lambda operators have not revealed nonspecific binding of adjacent CI dimers [14, 23, 24]. In these reports CI concentrations were limited to slightly more than 100 nM, while we used up to 500 nM CI in the present titrations. In addition, the free energy for non-specific binding of CI is -9.5 kcal/mol, which is at the very limit of sensitivity of the DNase footprinting assay, -9 kcal/mol [14]. Other experimental conditions may have interfered with the detection of non-specifically bound CI by footprinting, such as the presence of competitor DNA and divalent cations [14, 25]. Indeed, 3:1 ratios of unlabeled:labeled DNA dramatically reduced the binding of CI to DNA in control experiments (Fig 5).

Titration of competitor DNA reduced CI binding from 100 to 0% in the case of the OL1 and OL3 operators, and to 20% in the case of molecules containing the OR3 operator. In all cases, a plateau appears to separate two distinct declines of the CI-bound DNA fraction (Fig 5). We interpreted these as two species with either one or two CI dimers directly bound to DNA, respectively. We then performed FCS measurements using a CI mutant, D197G, which cannot interact with adjacent dimers. In this case, we only detected one complex which appears to correspond to DNA with just one CI dimer specifically bound to the operator.

### CI complexes grow larger than dodecamers

Our previous work [11] revealed that the dominant fraction of CI complexes securing loops are formed of 5–6 CI dimers with a volume of around 600 nm^3^. Again, we have found that the volume of the CI complex securing a loop increased with CI concentration (Fig 6B). 400 nM wild-type CI produced a fraction of complexes larger than those at 200 nM CI, and the sizes of almost all complexes grew further at 800 nM CI. In contrast, similar titrations of a mutant CI repressor, D197G, produced complexes composed mostly of 4 and rarely 6 dimers securing the majority of loops observed in samples prepared with either 200 or 400 nM protein. Interactions between the wild-type proteins appear to facilitate positioning and non-specific binding of adjacent dimers to create more extensive complexes that can further stabilize loops.

Thus our data show that semi-specific binding occurs with both O1 and O3 operators as CI concentration increases. This is a remarkable finding for two reasons. First, it shows that the closure of the lambda loop can be mediated by more dimers than can be accommodated specifically. Second, it shows that even a dimer bound at weak OR3 facilitates cooperative binding of another CI to an adjacent, non-specific site. Thus, additional CI might zipper DNA segments beyond OL1 and OR1 external to the OL-OR loop, or once OR3 in juxtaposition to OL3 via OL-OR looping becomes occupied, additional CI can zip together internal segments of the loop. Of course adjacent CI dimers in series like birds on a wire may toggle between alternative interactions within the series to produce dynamic changes in the interactions that secure the lambda loop, and this may be important to insure at least transient loop breakdown to expose CI for degradation if a switch to lysis is eminent. This underscores the previously suggested idea that semi-specific CI binding might play an important role in the dynamics of the lambda genetic switch [8].

The added semi-specific interactions might explain why the lambda lysogeny/lysis genetic switch has evolved to have an odd number of binding sites in each region participating in loop formation. By favoring additional pairwise, semi-specific interactions, the third operator in each region may favor the formation of a continuum of similarly bound states and maintain a high concentration of CI dimers near specific binding sites. The alternative possibilities for pairwise interactions would destabilize the repressive loop just enough to maintain the sensitivity of the switch to lysis. Ultimately, extended pair-wise interactions that include non-specific binding could be key to tight control of loop formation and breakdown. This, in turn, may suppress cell-to-cell variability, control transcriptional noise, and maintain lysogeny even at low CI concentrations [26].

## Supporting information

S1 Text Finding the optimal DNA and salt concentration for FCS measurements. (DOCX)

S2 Text Fitting strategy of FCS data with a two-stage, two-component diffusion model. (DOCX)

S1 Fig FCS data for OL1wild DNA fragments in the absence (black) or presence (red) of 500 nM mutant (D197G) CI protein. The autocorrelation function without protein was well fit by a single diffusing species (labeled DNA) but that in the presence of protein displayed extremely fast diffusion and was well fit by a single exponential component. Residual differences between the fits and the data are shown for no protein (center right) and 500 nM protein (bottom right). The correlation function of the free dye (blue) is also shown with its residual distribution (top right)**.**
(PPTX)

S2 Fig Data with which to determine the optimal DNA (a, c) and salt (KCl) concentrations (b, d) from measurements of the number of particles in the focal volume and the counts per particle.
(PPTX)

S3 Fig The dependence on protein concentration of the diffusion time of OL1wild DNA. (a) Data (black squares) were fit (red curve) using a single-phase binding model (between A and B, top left panel) or a bi-phasic binding model (among A, B and C, bottom left panel). The fitting which assumes bi-phasic behavior best captures the trend of the data. Analysis of the residual differences between the fitting and the data (b) shows non-random behavior (see areas in red circles) at low protein concentrations in the case of the single-phase binding model (black). However, the residual differences are random in the case of the two-phase binding model (red).
(PPTX)

S4 Fig Representative autocorrelation functions and the residuals for their fitting with one or two components with no protein (left), 250 nM (middle), or 500 nM CI (right). (PPTX)

S5 Fig Representative autocorrelation functions and the residuals for their fitting with one or two components in the presence of 150 (left) or 350 (right) nM CI. (PPTX)

S6 Fig A schematic diagram of the components required to fit the auto-correlation data accurately enough to avoid non-random error fluctuations. (PPTX)

S7 Fig Change in diffusion time for the OL1wild DNA in presence of increasing concentrations of D197G mutant protein. (PPTX)

S8 Fig The relative, first component amplitude in two-component plots (fraction of DNA with a single bound protein) displayed Hill-equation responses when DNA fragments containing the OL1 (black), OL3 (red) and OR3 (blue) operators were titrated with WT (left) and mutant (right) lambda repressors. The data in the right panel were taken directly from Fig 2.
(PPTX)

S9 Fig Representative nanographs of wild-type CI complexes securing 400 bp DNA loops. The CI complexes increased in size as the CI concentration was raised.
(PPTX)

S1 Table Names and schematic compositions of DNA constructs used in FCS measurements. In the descriptions, “ALEX488” indicates the fluorophore label, “5” indicates the number of base pairs separating ALEX-488 from the specific lambda repressor operator (OL3, OR3, or OL1), NS38 indicates a sequence of 38 base pairs of non-specific DNA with the wild type (WILD), or a random, GC-enriched (RNDM_GC) sequence.
(DOC)

S2 Table Diffusion times of the protein-DNA complexes for both the WT and mutant CI proteins. (DOC)

S3 Table Dissociation constants for the WT CI and D197G mutant with operator DNAs were obtained by fitting the data to the Hill equation. Note that the activities of the protein preparations were not determined but were likely to be 50% or higher. In addition, previously measured specific and non-specific affinities of repressor proteins were shown to decrease by one to two orders of magnitude as the DNA was shortened (Senear DF, Batey R. *Biochemistry*. 1991; **30**(27):6677–88; Winter RB, von Hippel PH. *Biochemistry*. 1981; **20**(24):6948–60). Thus, the dissociation constants above should be considered upper limits
(DOC)
