# Supplementary material for: Specifically bound lambda repressor dimers promote adjacent non-specific binding
Source: PLoS One. 2018 Apr 2;13(4):e0194930. doi: 10.1371/journal.pone.0194930 (PMC5880393; doi:10.1371/journal.pone.0194930)
Supplement: S1 Text — (DOCX) [file pone.0194930.s001.docx]

**Supplemental Text**

**Specifically bound Lambda repressor protein promotes adjacent non-specific binding**

Suparna Sarkar-Banerjee^1,#a^, Sachin Goyal^2^, Ning Gao^3^, John Mack^3^, Benito Thompson^3^, David Dunlap^3^, Krishnananda Chattopadhyay^4,^[[1]](#footnote-1)^^ and Laura Finzi^3,*^

^1^ Protein Folding and Dynamics Laboratory, Structural Biology and Bioinformatics Division, CSIR-Indian Institute of Chemical Biology, 4, Raja S. C. Mullick Road, Kolkata 700032, India.

^2^ Department of Mechanical Engineering, University of California, Merced, CA 95343, USA.

^3^ Physics Department, Emory University, Atlanta, GA 30322, USA.

^#a^ Department of Integrative Biology and Pharmacology, McGovern Medical School at UTHealth, Houston, Texas 77030, USA.

^*^Corresponding authors:

[lfinzi@emory.edu](mailto:lfinzi@emory.edu) (LF)

[krishnanandac@yahoo.com](mailto:krishnanandac@yahoo.com) (KC)

## S1 Text. Finding the optimal DNA and salt concentration for FCS measurements

DNA constructs for FCS featured Alexa-488 at the free end followed by 5 base pairs, the operator sequence, and finally 38 base pairs of either wild-type flanking sequence, or a random, GC-enriched sequence. Representative auto-correlation functions are shown in S1 Fig. for the OL1wild DNA construct (Table S1) alone or with mutant (D197G) CI protein. The correlation function obtained for free Alexa-488 was well fit using a single diffusive component model (S1 Fig., left panel, blue triangles) and the goodness of the fit was verified by the randomness of the residual distribution (Fig. S1, bottom right panel). S2 Fig. shows the dependence of the number of simultaneously detected particles (N) and counts per particle on the concentration of labeled DNA (panels *a* and *c*, respectively). As expected, *N* increases with increasing DNA concentration. We chose to use a concentration of 100 nM DNA for subsequent experiments.

In order to compare our FCS results with other work recently published on CI-mediated DNA looping [1, 2], a 10 mM Tris-HCl buffer at pH 7.4 supplemented with 200 mM KCl was used. However, the index of refraction and viscosity of the solution vary with salt concentration and can cause optical aberration which significantly affects the resolution and sensitivity of FCS detection [3]. Therefore, we also performed a titration, similar to that carried out for DNA, in which we monitored *N* and counts per particle as the concentration of KCl in the buffer was varied from 0 to 600 mM. Although there was an increase in the number of particles *N* (and a decrease in the counts per particle) (S2b and S2d Figs.), as expected from previously reported effects of the index of refraction of the solution on these two parameters [3, 4], we obtained sufficient counts from the FCS experiments to be able to use the previously published buffer conditions in our experiments.

**Supporting Text References**

1. Manzo C, Zurla C, Dunlap DD, Finzi L. The Effect of Nonspecific Binding of Lambda Repressor on DNA Looping Dynamics. Biophysical Journal. 2012;103(8):1753-61. doi: 10.1016/j.bpj.2012.09.006. PubMed PMID: WOS:000310100400017.

2. Zurla C, Manzo C, Dunlap D, Lewis DEA, Adhya S, Finzi L. Direct demonstration and quantification of long-range DNA looping by the λ bacteriophage repressor. Nucleic Acids Research. 2009;37(9):2789-95. doi: 10.1093/nar/gkp134.

3. Chattopadhyay K, Saffarian S, Elson EL, Frieden C. Measuring Unfolding of Proteins in the Presence of Denaturant Using Fluorescence Correlation Spectroscopy. Biophysical Journal. 88(2):1413-22. doi: 10.1529/biophysj.104.053199.

4. Hess ST, Webb WW. Focal Volume Optics and Experimental Artifacts in Confocal Fluorescence Correlation Spectroscopy. Biophysical Journal. 2002;83(4):2300-17. doi: <http://dx.doi.org/10.1016/S0006-3495(02)73990-8>.

1. [↑](#footnote-ref-1)
