# Supplementary material for: Specifically bound lambda repressor dimers promote adjacent non-specific binding
Source: PLoS One. 2018 Apr 2;13(4):e0194930. doi: 10.1371/journal.pone.0194930 (PMC5880393; doi:10.1371/journal.pone.0194930)
Supplement: S2 Text — (DOCX) [file pone.0194930.s002.docx]

**Supplemental Text**

**Specifically bound Lambda repressor protein promotes adjacent non-specific binding**

Suparna Sarkar-Banerjee^1,#a^, Sachin Goyal^2^, Ning Gao^3^, John Mack^3^, Benito Thompson^3^, David Dunlap^3^, Krishnananda Chattopadhyay^4,^[[1]](#footnote-1)^^ and Laura Finzi^3,*^

^1^ Protein Folding and Dynamics Laboratory, Structural Biology and Bioinformatics Division, CSIR-Indian Institute of Chemical Biology, 4, Raja S. C. Mullick Road, Kolkata 700032, India.

^2^ Department of Mechanical Engineering, University of California, Merced, CA 95343, USA.

^3^ Physics Department, Emory University, Atlanta, GA 30322, USA.

^#a^ Department of Integrative Biology and Pharmacology, McGovern Medical School at UTHealth, Houston, Texas 77030, USA.

^*^Corresponding authors:

[lfinzi@emory.edu](mailto:lfinzi@emory.edu) (LF)

[krishnanandac@yahoo.com](mailto:krishnanandac@yahoo.com) (KC)

# **S2 Text. Fitting strategy of FCS data with a two-stage two-component diffusion model**

The FCS data were initially fit using a single component diffusion model (Equation 1) for all protein concentrations. Fig. S3a shows the values of *τ*_D_ plotted with increasing CI concentrations for the representative example of the OL1wild DNA construct. The data were first fit, assuming monophasic binding between two species (A and B in S3a Fig., top), using the dose-response model of Origin 8 Pro (OriginLab Corp. MA). This fitting however showed non-random behavior at the initial portion of the residual distribution (shown in the red circles in S3b Fig.). In contrast, the fit was significantly better assuming a biphasic binding of CI to DNA using the bidose-response model of Origin 8 Pro. In this case, three species (A, B, and C in S3a Fig., bottom) would form with increasing CI concentration. The random distribution of the residuals supports the goodness of this fit (S3b Fig.). This analysis provided initial indication that a single-component model was not always adequate to interpret the FCS data obtained at different protein concentrations.

Furthermore, fits of the auto-correlation functions obtained in the absence of protein, at 250 nM and at saturating CI concentration (500nM), were well fit by a single component diffusion model indicated by random fluctuations in plots of the residuals (S4 Fig., middle row). The addition of a second fitting component introduced non-random fluctuations in plots of the residuals. This indicates that the fitting model is not optimal (S4 Fig., bottom row).

In contrast, a one-component model did not adequately fit the autocorrelation data for intermediate protein concentrations between 0 and 250 nM or between 250 and 500 nM. S5 Fig. shows the residual plots for representative intermediate concentrations, 125 or 350 nM. For these data, the amplitudes of a two-component model with *τ_D_* values established from the one-component fit regions on either side were fitted. For example, for the first transition, the *τ_D_* values of two diffusion components obtained with 0 nM and 250 nM CI were set as *τ_D1_* and *τ_D2_*, and the values of *a_1_* and *a_2_* were fit. A similar strategy was used for the second transition to determine *a_2_* and *a_3_*. This strategy produced close fits of the auto-correlation data and random fluctuations in the residual plots and is graphically summarized in S6 Fig.

1. [↑](#footnote-ref-1)
