# Supplementary figures and images for: Specifically bound lambda repressor dimers promote adjacent non-specific binding
Source: PLoS One. 2018 Apr 2;13(4):e0194930. doi: 10.1371/journal.pone.0194930 (PMC5880393; doi:10.1371/journal.pone.0194930)

## Slide 1
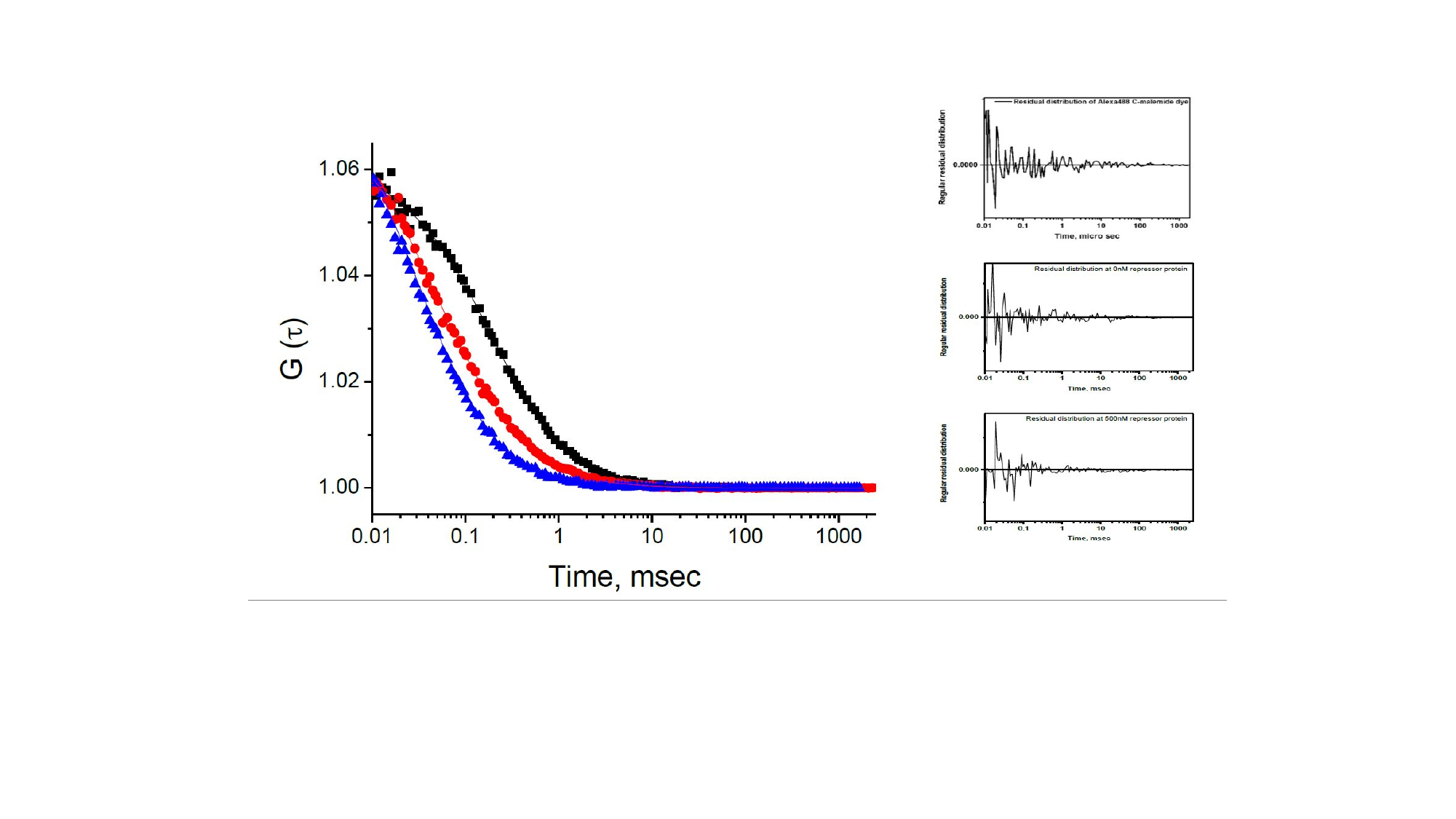

Supplement: S1 Fig — The autocorrelation function without protein was well fit by a single diffusing species (labeled DNA) but that in the presence of protein displayed extremely fast diffusion and was well fit by a single exponential component. Residual differences between the fits and the data are shown for no protein (center right) and 500 nM protein (bottom right). The correlation function of the free dye (blue) is also shown with its residual distribution (top right). (PPTX) [file pone.0194930.s003.pptx]

## Slide 1
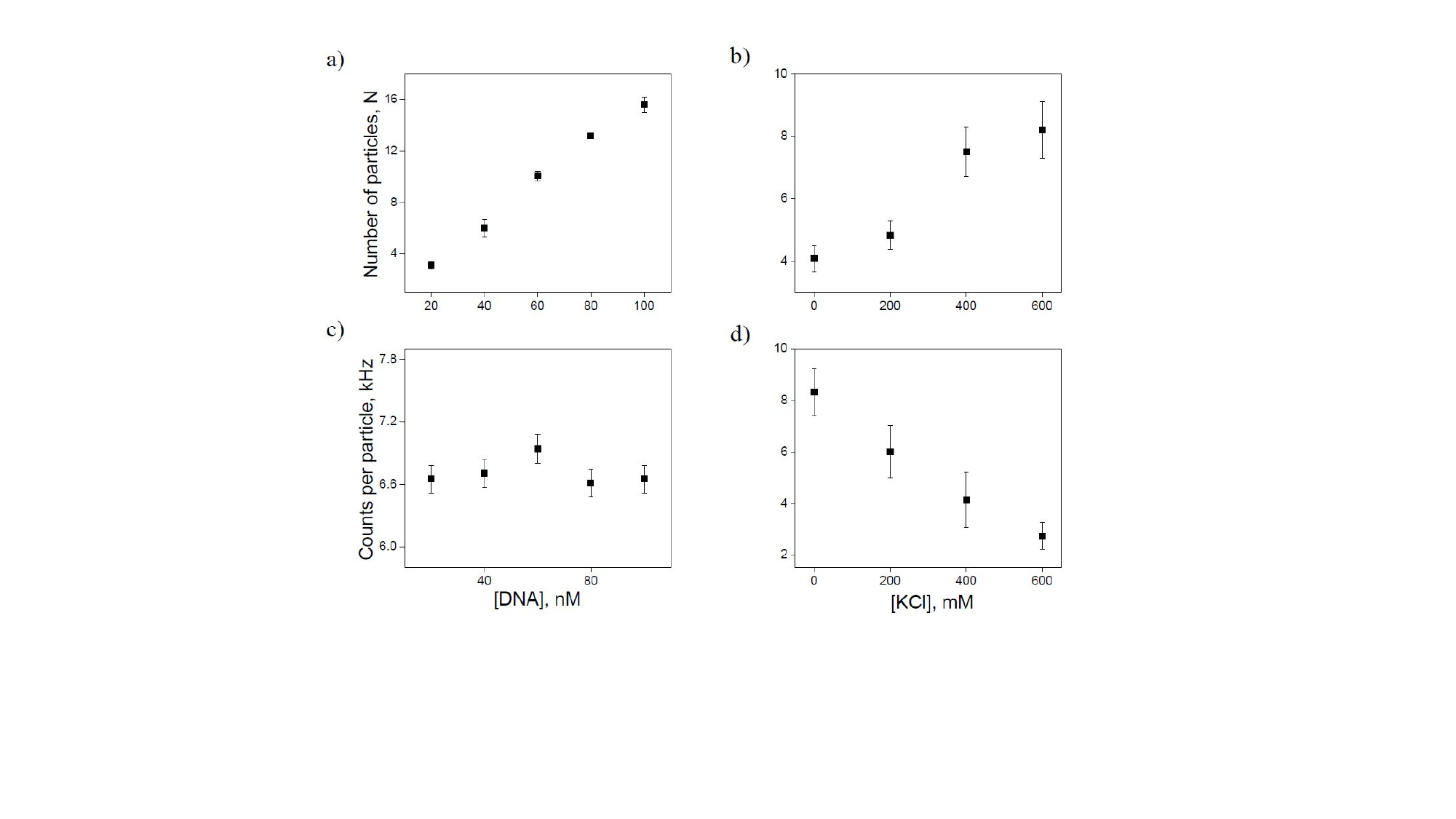

Supplement: S2 Fig — Data with which to determine the optimal DNA (a, c) and salt (KCl) concentrations (b, d) from measurements of the number of particles in the focal volume and the counts per particle. (PPTX) [file pone.0194930.s004.pptx]

## Slide 1
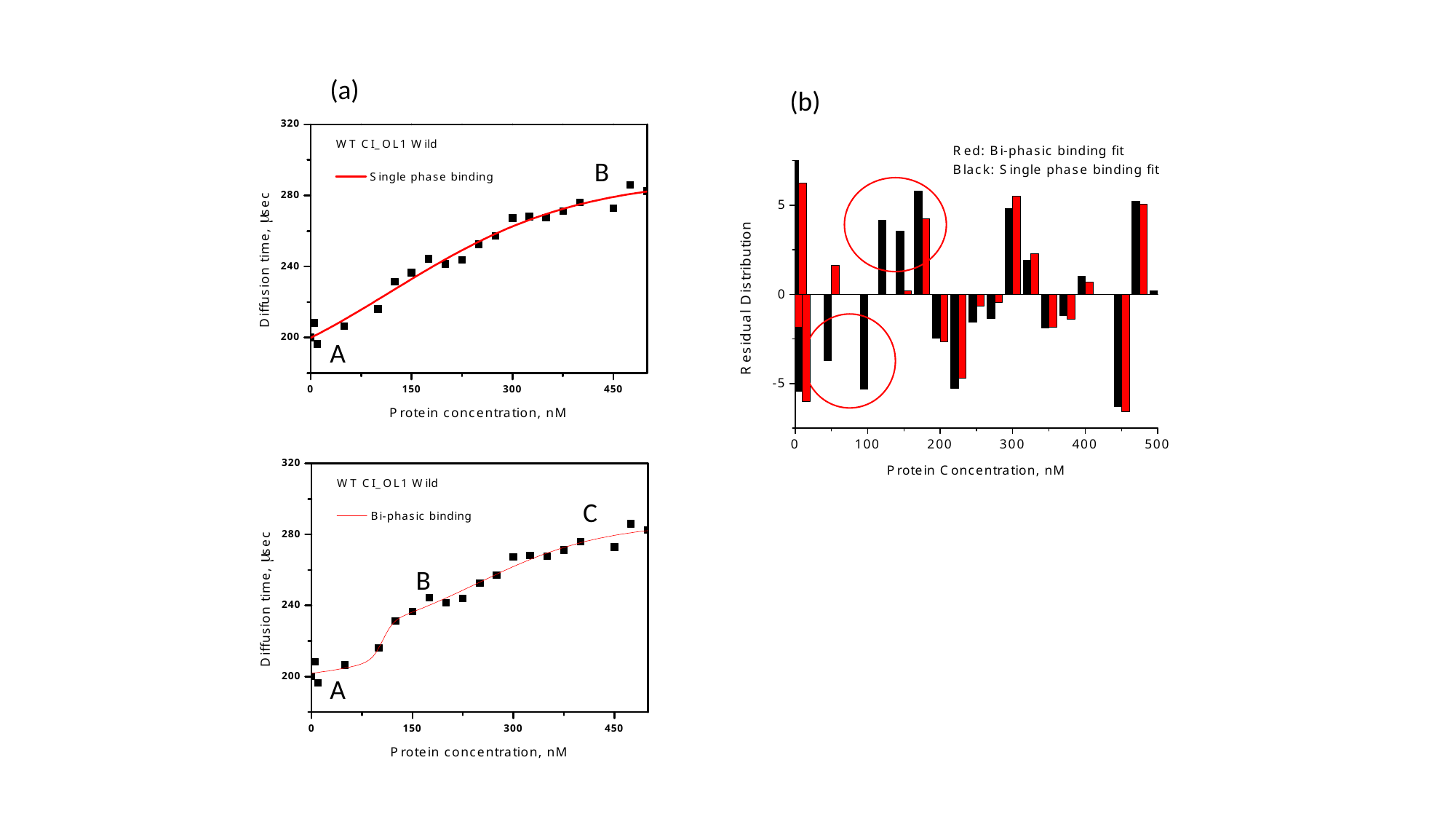

(a)
(b)
B
A
C
B
A

Supplement: S3 Fig — (a) Data (black squares) were fit (red curve) using a single-phase binding model (between A and B, top left panel) or a bi-phasic binding model (among A, B and C, bottom left panel). The fitting which assumes bi-phasic behavior best captures the trend of the data. Analysis of the residual differences between the fitting and the data (b) shows non-random behavior (see areas in red circles) at low protein concentrations in the case of the single-phase binding model (black). However, the residual differences are random in the case of the two-phase binding model (red). (PPTX) [file pone.0194930.s005.pptx]

## Slide 1
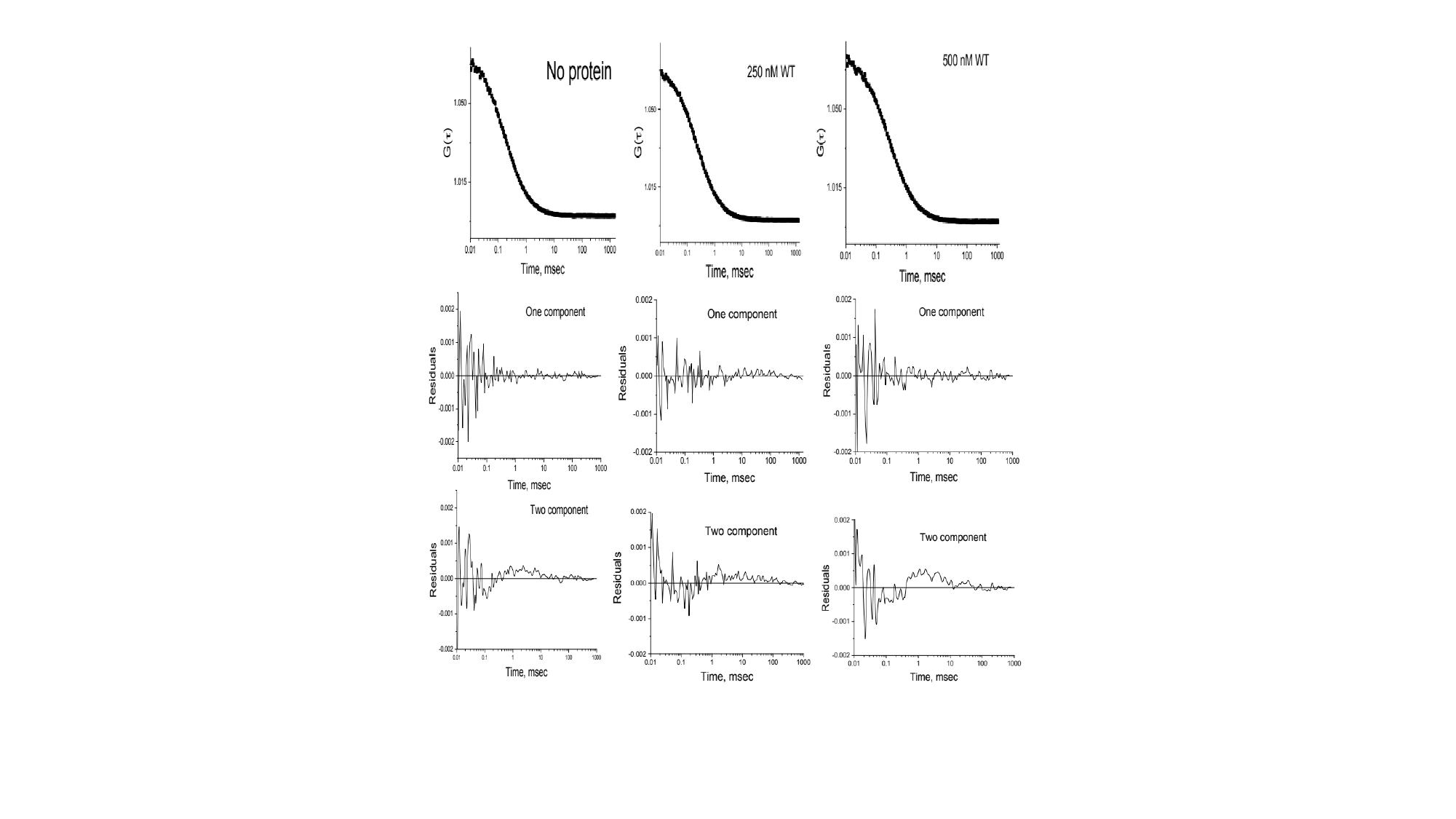

Supplement: S4 Fig — (PPTX) [file pone.0194930.s006.pptx]

## Slide 1
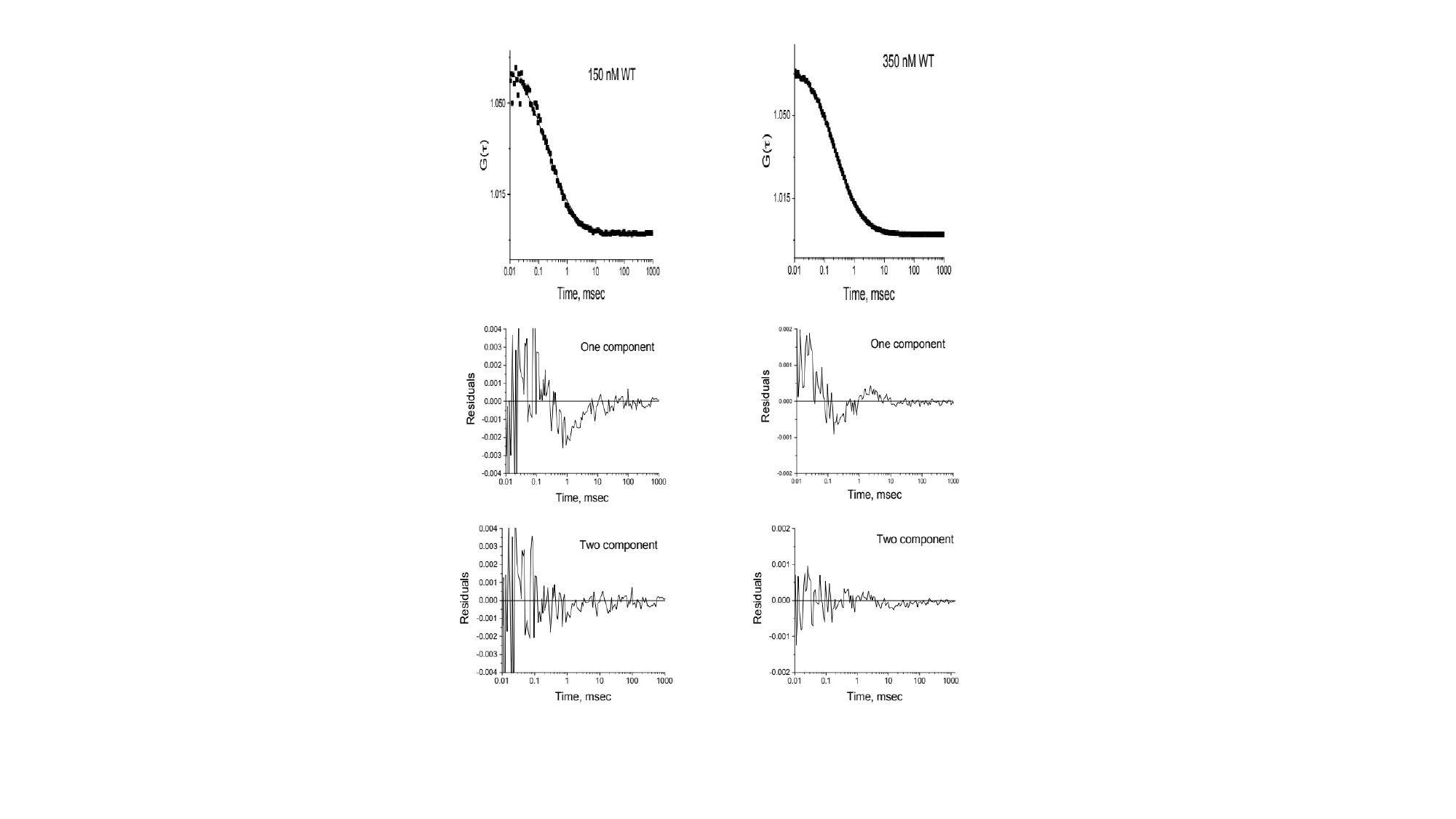

Supplement: S5 Fig — (PPTX) [file pone.0194930.s007.pptx]

## Slide 1
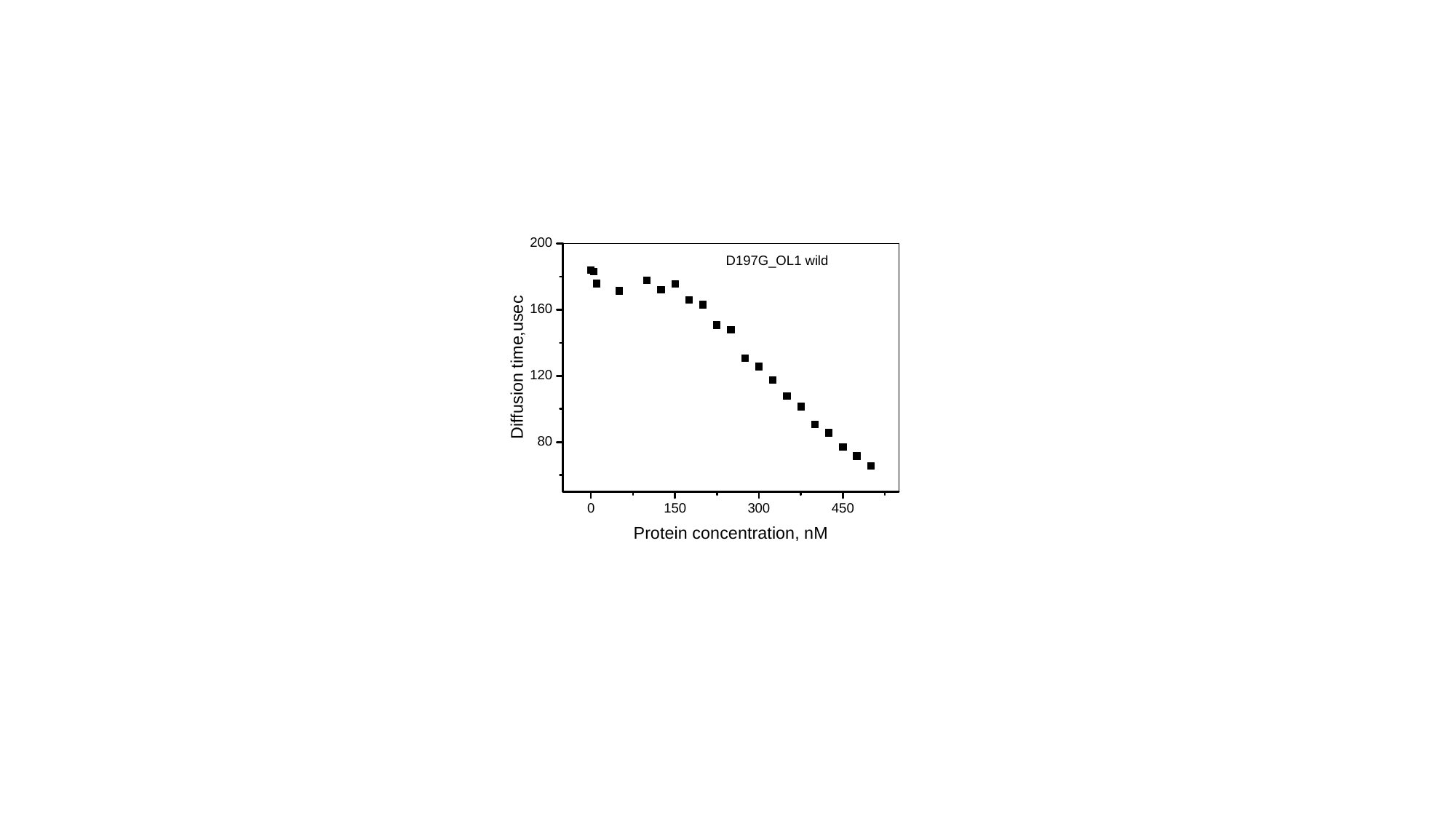

Supplement: S7 Fig — (PPTX) [file pone.0194930.s009.pptx]

## Slide 1
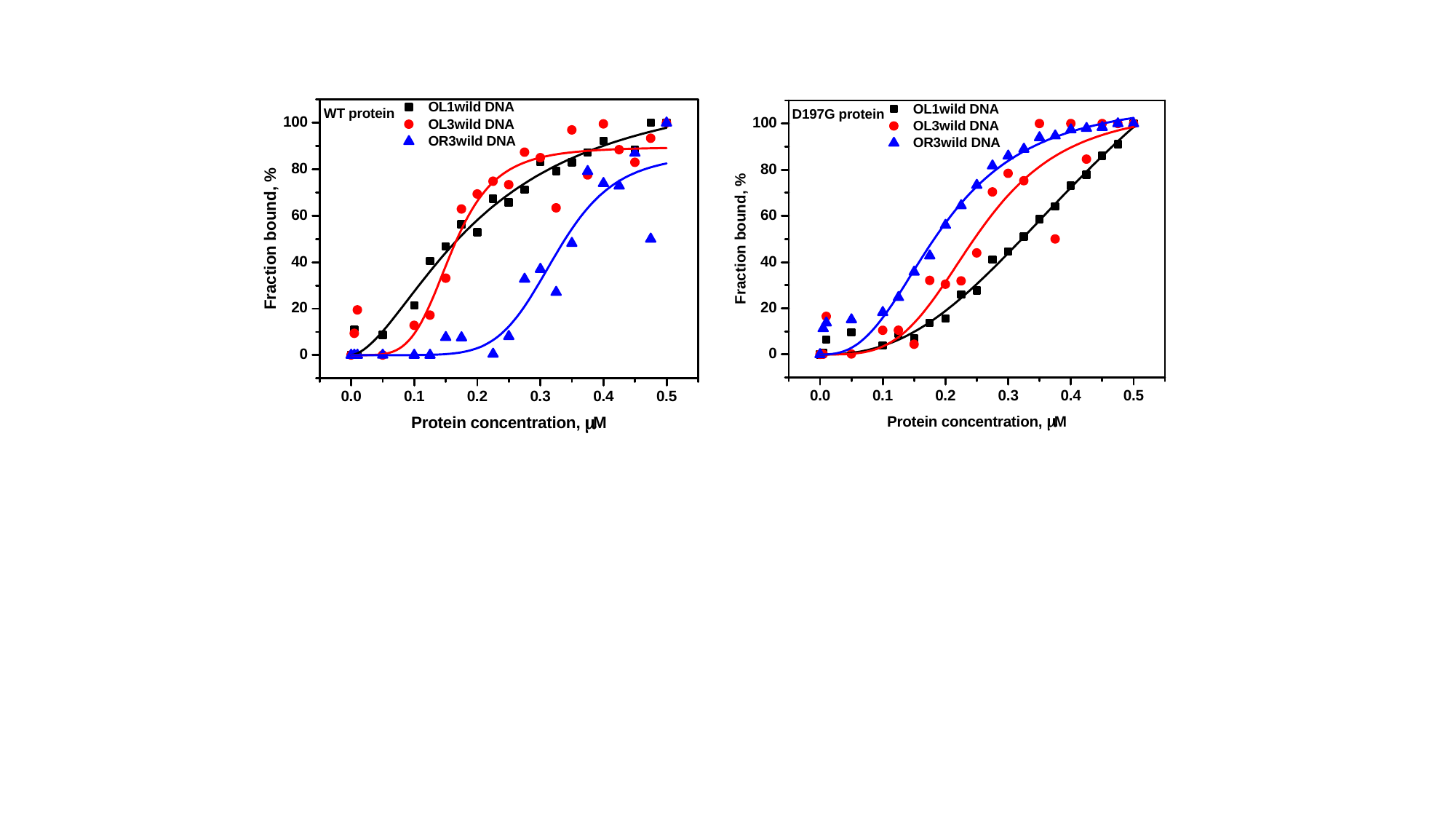

Supplement: S8 Fig — The data in the right panel were taken directly from Fig 2. (PPTX) [file pone.0194930.s010.pptx]

## Slide 1
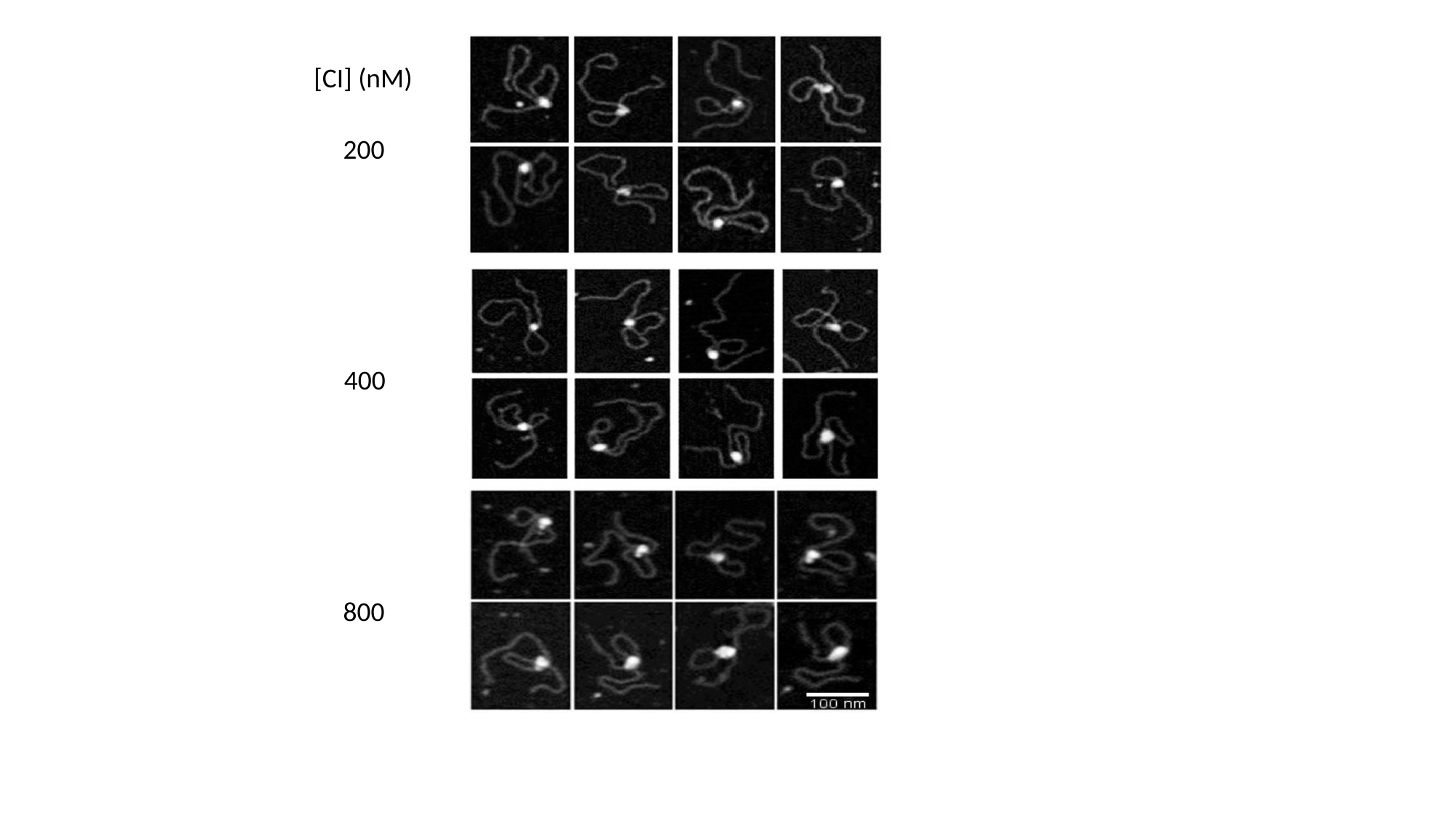

[CI] (nM)
200
400
800

Supplement: S9 Fig — The CI complexes increased in size as the CI concentration was raised. (PPTX) [file pone.0194930.s011.pptx]
