# Supplementary material for: Specifically bound lambda repressor dimers promote adjacent non-specific binding
Source: PLoS One. 2018 Apr 2;13(4):e0194930. doi: 10.1371/journal.pone.0194930 (PMC5880393; doi:10.1371/journal.pone.0194930)
Supplement: S6 Fig — (PPTX) [file pone.0194930.s008.pptx]

## Slide 1
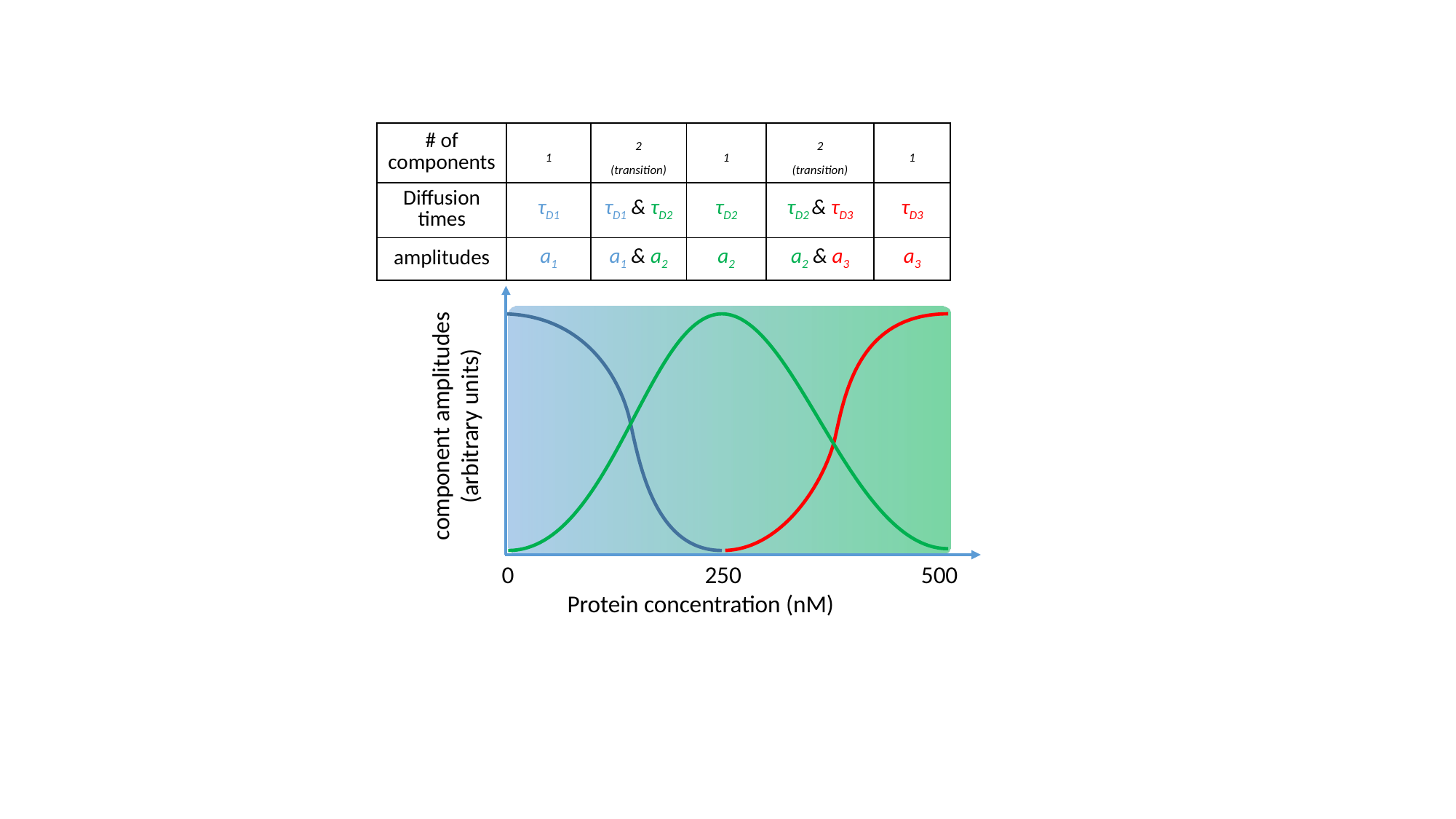

| # of components | 1 | 2 (transition) | 1 | 2 (transition) | 1 |
| --- | --- | --- | --- | --- | --- |
| Diffusion times | τD1 | τD1 & τD2 | τD2 | τD2 & τD3 | τD3 |
| amplitudes | a1 | a1 & a2 | a2 | a2 & a3 | a3 |
component amplitudes (arbitrary units)
0 250 500
 Protein concentration (nM)
