# Supplementary material for: Specifically bound lambda repressor dimers promote adjacent non-specific binding
Source: PLoS One. 2018 Apr 2;13(4):e0194930. doi: 10.1371/journal.pone.0194930 (PMC5880393; doi:10.1371/journal.pone.0194930)
Supplement: S1 Table — In the descriptions, “ALEX488” indicates the fluorophore label, “5” indicates the number of base pairs separating ALEX-488 from the specific lambda repressor operator (OL3, OR3, or OL1), NS38 indicates a sequence of 38 base pairs of non-specific DNA with the wild type (WILD), or a random, GC-enriched (RNDM_GC) sequence. (DOC) [file pone.0194930.s012.doc]

# **Table S1**

| **Name** | **Description** | **Sequence** |
| --- | --- | --- |
| OL3-wild | ALEX488_5_OL3_NS38_WILD | 5'-Alexa488-TAATTTATCACCGCAGATGGTTATCTGTATGTTTTTTATATGAATTTATTTTTTGCAGGG |
| OL3-RndmGC | ALEX488_5_OL3_NS38_RNDM_GC | 5'-Alexa488-TAATTTATCACCGCAGATGGTTGCCCATCCTGGGTCGGCACATCGGTGTGGGTTAGGGCC |
| OL1-wild | NS38_WILD_OL1_5_ALEX488 | CCTTCATGGTGGTCAGTGCGTCCTGCTGATGTGCTCAGTATCACCGCCAGTGGTATTTAT-Alexa488-3' |
| OL1-RndmGC | NS38_RNDM_GC_OL1_5_ALEX488 | GCCCATCCTGGGTCGGCACATCGGTGTGGGTTAGGGCCTATCACCGCCAGTGGTATTTAT-Alexa488-3' |
| OR3-wild | NS38_WILD_OR3_5_ALEX488 | GTGTTAATGGTTTCTTTTTTGTGCTCATACGTTAAATCTATCACCGCAAGGGATAAATAT-Alexa488-3' |
| OR3-RndmGC | NS38_RNDM_GC_OR3_5_ALEX488 | GCCCATCCTGGGTCGGCACATCGGTGTGGGTTAGGGCCTATCACCGCAAGGGATAAATAT-Alexa488-3' |
