# Supplementary material for: Specifically bound lambda repressor dimers promote adjacent non-specific binding
Source: PLoS One. 2018 Apr 2;13(4):e0194930. doi: 10.1371/journal.pone.0194930 (PMC5880393; doi:10.1371/journal.pone.0194930)
Supplement: S2 Table — (DOC) [file pone.0194930.s013.doc]

# **Table S2**

| DNA | τ_D wild-type_ (μs) | τ_D_ _D197G_ (μs) |
| --- | --- | --- |
| OL1wild | 280 ± 0.02 | 71.49 ± 0.05 |
| OL3wild | 290 ± 0.005 | 230 ± 0.03 |
| OR3wild | 300 ± 0.01 | 41 ± 0.01 |
| Free dye | 30 ± 0.002 | 30 ± 0.002 |
