# Supplementary material for: Specifically bound lambda repressor dimers promote adjacent non-specific binding
Source: PLoS One. 2018 Apr 2;13(4):e0194930. doi: 10.1371/journal.pone.0194930 (PMC5880393; doi:10.1371/journal.pone.0194930)
Supplement: S3 Table — Note that the activities of the protein preparations were not determined but were likely to be 50% or higher. In addition, previously measured specific and non-specific affinities of repressor proteins were shown to decrease by one to two orders of magnitude as the DNA was shortened (Senear DF, Batey R. Biochemistry. 1991; 30(27):6677–88; Winter RB, von Hippel PH. Biochemistry. 1981; 20(24):6948–60). Thus, the dissociation constants above should be considered upper limits (DOC) [file pone.0194930.s014.doc]

# **Table S3**

| DNA | *K_d_*, M (WT CI) | *K_d_*, M (D197G) |
| --- | --- | --- |
| OL1 wild | 2.09 (± 0.03) X 10^-7^ | 4.99 (± 0.09) X 10^-7^ |
| OL3 wild | 1.59 (± 0.01) X 10^-7^ | 2.55 (± 0.02) X 10^-7^ |
| OR3 wild | 3.24 (± 0.02) X 10^-7^ | 1.96 (± 0.01) X 10^-7^ |
